# RNA-Seq profiling of deregulated miRs in CLL and their impact on clinical outcome

**DOI:** 10.1038/s41408-019-0272-y

**Published:** 2020-01-13

**Authors:** Gurvinder Kaur, Vivek Ruhela, Lata Rani, Anubha Gupta, Krishnamachari Sriram, Ajay Gogia, Atul Sharma, Lalit Kumar, Ritu Gupta

**Affiliations:** 1 Laboratory Oncology Unit, Dr. B.R.A.IRCH, All India Institute of Medical Sciences, New Delhi, India; 2 Department of Computational Biology, Indraprastha Institute of Information Technology-Delhi (IIIT-D), Delhi, India; 3 SBILab, Department of Electronics and Communication Engineering, Indraprastha Institute of Information Technology-Delhi (IIIT-D), Delhi, India; 4 Department of Medical Oncology, Dr. B.R.A.IRCH, All India Institute of Medical Sciences, New Delhi, India

**Keywords:** Cancer genetics, Translational research

## Abstract

Abnormal expression patterns of regulatory small non-coding RNA (sncRNA) molecules such as microRNAs (miRs), piwi-interacting RNAs (piRNAs), and small nucleolar RNAs (snoRNAs) play an important role in the development and progression of cancer. Identification of clinically relevant sncRNA signatures could, therefore, be of tremendous translational value. In the present study, genome-wide small RNA sequencing identified a unique pattern of differential regulation of eight miRs in Chronic Lymphocytic Leukemia (CLL). Among these, three were up-regulated (miR-1295a, miR-155, miR-4524a) and five were down-regulated (miR-30a, miR-423, miR-486*, let-7e, and miR-744) in CLL. Altered expression of all these eight differentially expressed miRs (DEMs) was validated by RQ-PCR. Besides, seven novel sequences identified to have elevated expression levels in CLL turned out to be transfer RNA (tRNA)/piRNAs (piRNA-30799, piRNA-36225)/snoRNA (SNORD43) related. Multivariate analysis showed that miR-4524a (HR: 1.916, 95% CI: 1.080–3.4, *p* value: 0.026) and miR-744 (HR: 0.415, 95% CI: 0.224–0.769, *p* value: 0.005) were significantly associated with risk and time to first treatment. Further investigations could help establish the scope of integration of these DEM markers into risk stratification designs and prognostication approaches for CLL.

## Introduction

Chronic Lymphocytic Leukemia (CLL) is a clinically heterogeneous malignancy where a large molecular inter-individual heterogeneity is observed which is fundamentally governed by differences in the underlying genetic vulnerabilities of individual cases^1^. Congruent molecular and pathological studies have identified a number of potential genomic biomarkers for prognosis or response to therapy in CLL. The most persistently observed somatic copy number variations (CNVs) with prognostic significance in CLL include del(13q14), del(11q22.3), del(17p), trisomy 12, amp(8q24.21), amp(3q26.32) and del(8p)^2^. Recurrent mutations among genes believed to act as putative drivers of CLL such as *TP53, SF3B1, NOTCH1, MYD88, ATM*, *SAMHD1*, *NRAS*, and *BIRC3* have also been shown to exhibit a significant prognostic association^3^.

Recent genomic studies using parallel high throughput technologies like Next-generation sequencing (NGS) and microarrays have revealed that the molecular heterogeneity of CLL is further complicated by alterations in gene expression patterns and epigenetic regulatory events; and abundance of long noncoding RNAs (lncRNA) and small noncoding RNAs (sncRNAs) such as microRNAs (miRs), tRNA, piRNA, snoRNA, etc^4,5^. A plethora of studies on transcriptional profiling of miRs have identified a variety of differentially expressed miRs (DEMs) in CLL^6–10^. In the landmark study of Calin et al., a 13 miR signature was reported in CLL patients with high Zeta-chain-associated protein kinase 70 (ZAP70) expression and unmutated immunoglobulin heavy chain variable region gene (IGHV) status^6^. Differential expression of various miRNAs including miR-15a, miR-16, miR-29a/b/c, miR-223 and miR-150 have been consistently reported to be associated with well established prognostic factors such as *IGHV* status, ZAP70/CD38 expression, β2 microglobulin levels and disease progression in CLL^11^. A few studies have identified karyotype specific miR signatures in CLL that could discriminate patients harboring del(17p), del(11q), del(13q), trisomy 12, and normal karyotype^12,13^. In patients with commonly encountered del(13q14), the co-localized tumor suppressive miR-15a and miR-16-1 get deleted, leading to increased *BCL-2* expression that facilitates initiation of CLL^14^. Del(11q) is associated with co-deletion of miR-34b/c clusters^15^ as well as elevated levels of miR-769-5p and miR-338-3p^16^ while trisomy 12 has been shown to be associated with up-regulation of miR-181a and down-regulation of miR-155, miR-148a, and miR-483-5p in CLL^12,16^. In poor prognostic subgroup with del(17p), differential regulation of various miRNAs such as miR-34a, miR-29b/c, miR-17-5p, miR-223, miR-150, miR-181, miR-33b, miR-96, and miR-21 has been observed^12,17^. Owing to the noteworthy prognostic potential of miRs, cumulative prognostic scores in combination with other prognostic factors have also been proposed in CLL^18,19^.

Keeping in view the growing diverse miR repertoire, their immense translational potential and advances in technology for their detection, we have undertaken this study and sequenced whole small RNA transcriptome in CLL. We have also co-analyzed genome-wide gene expression profiles to gain a deeper bimodal insight into the CLL miRnome circuitry and its mechanistic functional pathways. This study has demonstrated for the first time, unique patterns of DEMs, targets and deregulated piRNAs and snoRNAs related molecules in CLL. Further analysis has revealed significant impact of specific DEMs on clinical outcome in CLL.

## Materials and methods

### Subjects

CLL patients diagnosed as per the diagnostic criteria of the International Workshop on Chronic Lymphocytic Leukemia-sponsored Working Group^20^ and 10 age-matched (median age: 58.5 years; range: 55–61 years) healthy controls (5 males and 5 females) were enrolled. The demographic, clinical and laboratory based details of the cases evaluated for different sets of experiments are provided in Table 1. The study was conducted in accordance with the Declaration of Helsinki guidelines. Ethical clearance for the study was obtained from the institute’s ethics committee and written informed consent was obtained from all the participants.

**Table 1:** Baseline demographic, laboratory, and clinical characteristics of CLL patients as per different experimental cohorts.

Parameter	NGS (*n* = 28) Numbers (%)	Gene Expression Array (*n* = 21) Numbers (%)	RQ-PCR (*n* = 89) Numbers (%)
Gender			
Male	21 (75%)	16 (76%)	68 (76.5%)
Female	07 (25%)	05 (24%)	21 (23.5%)
Median age	60	60	60
≤65 years	20 (71.4%)	16 (76.2%)	69 (77.5%)
>65 years	08 (28.6%)	05 (23.8%)	20 (22.5%)
Rai stage			
Stage 0/I/II	04/06/07	04/08/09	15/14/28
Stage III/IV	05/06	−/−	13/19
Beta2 Microglobulin*			
≤3.5	2 (7.1%)	6 (30%)	15 (17.2%)
>3.5	26 (92.9%)	14 (70%	72 (82.8%)
IGHV mutational status**			
Mutated	10 (35.7%)	10 (48%)	47 (56%)
Unmutated	18 (64.3%)	11 (52%)	37 (44%)
Genetic abnormality***			
No abnormality	09 (32%)	08 (42%)	34 (40.5%)
Del (13q)+	07 (25%)	04 (21%)	22 (26.2%)
Del (11q)+	07 (25%)	03 (16%)	07 (8.3%)
Del (17p)+	01 (4%)	02 (10.5%)	14 (16.7%)
Trisomy12	04 (14%)	02 (10.5%)	07 (8.3%)

*Beta2 Microglobulin data was available for 20/21 and 87/89 patients of Gene expression (GE) array and RQ-PCR cohorts respectively. **IGHV mutational status was available for 84/89 patients of RQ-PCR cohort. ***Genetic aberrations data was available for 19/21 and 84/89 patients of GE array and RQ-PCR cohort respectively

### Genome-wide miR sequencing by NGS

Total RNA was extracted from the Mononuclear cells (MNCs) of CLL patients and MACS sorted CD19 + B cells (cat no. 130050301, Milteneyi Biotech, Germany) of healthy controls using the miRVana kit (Thermofisher Scientific, MA, USA). The samples having RNA Integrity Number (RIN) ≥ 7 were processed further. Small RNA libraries were generated for 28 CLL cases and 2 pooled healthy controls (pooled from 5 males and 5 females) using the “TruSeq small RNA sample preparation kit” (Illumina, San Diego, CA, USA) as per the manufacturer’s recommendations. The libraries with 76 nucleotide inserts were subsequently sequenced on NextSeq 500 (Illumina).

### RNA-Seq pipeline for analysis of NGS data

**Fig. 1:**
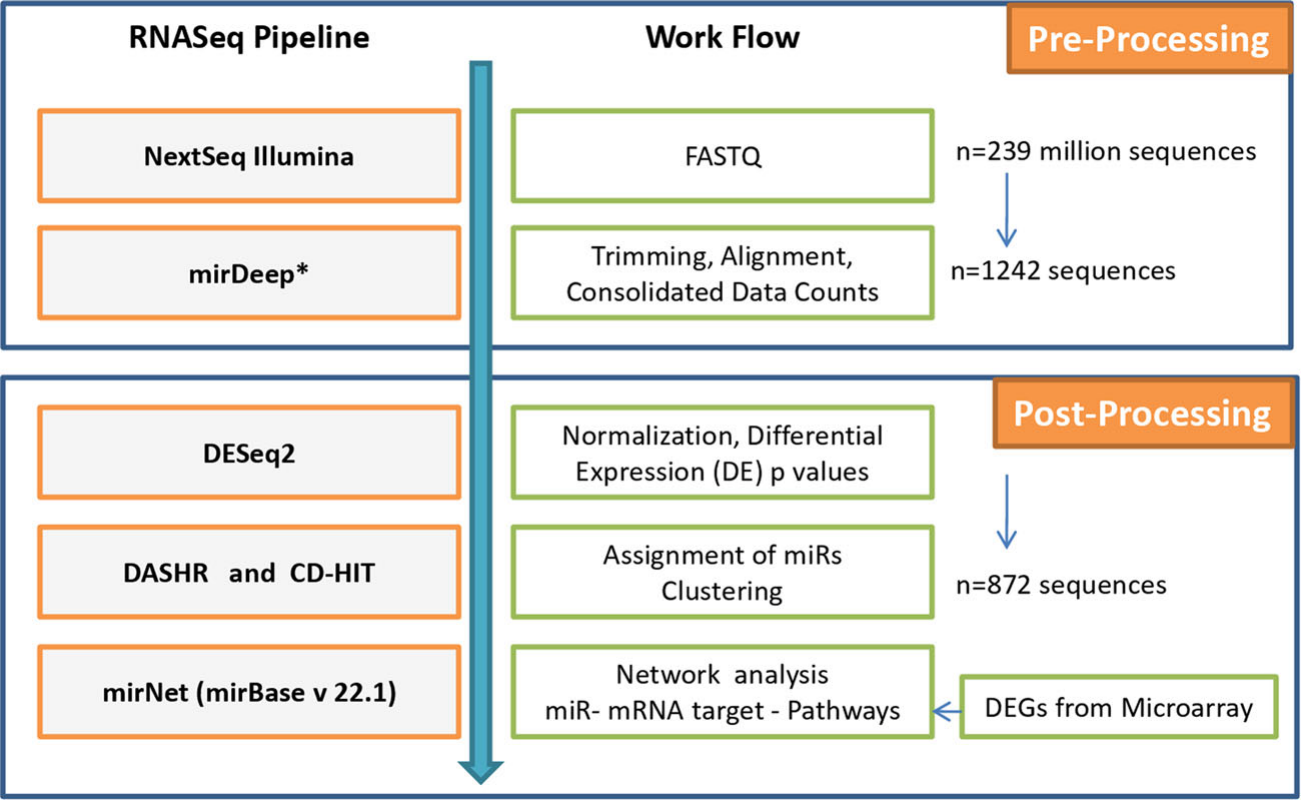
Bioinformatics workflow for the processing and analysis of RNASeq data.

FASTQ files as obtained from RNA-Seq experiments were further analyzed with the bioinformatics pipeline developed in house which was pre-validated on publicly available published data on acute lymphoblastic leukemia (ALL)^21^. A schematic representation of the RNA-Seq analysis pipeline and related workflow is shown in Fig. 1. The base quality of the raw reads (>Q30) was initially checked with java based FastQC developed by Babraham Bioinformatics (https://www.bioinformatics.babraham.ac.uk/projects/fastqc/). This was followed by adapter trimming and sequence alignment with GRCh37 human genome assembly database using miRDeep* (https://sourceforge.net/projects/miRdeepstar/) and miRBase v22.1^22^. Custom python script was used to compute consolidated count matrix from.result files obtained from miRDeep* and the duplicates were merged. The unaligned potential novel miRs were clustered with CD-HIT^23^ (http://weizhongli-lab.org/cdhit_suite/cgi-bin/index.cgi?cmd=cd-hit) and their unique IDs were generated. Sequence annotations of potential novel miRs were ascertained with DASHR (http://www.lisanwanglab.org/DASHR/smdb.php#tabSearch)^24^. The trimmed data obtained from miRDeep* was further processed with Bioconductor DESeq2 (https://bioconductor.org/packages/release/bioc/html/DESeq2.html)^25^ where the consolidated count data were normalized and DEMs were identified along with corresponding Wald p statistic and Benjamini-Hochberg adjusted *p* values to avoid false discovery rates.

The miRs with adjusted *p* values < 0.05, and fold change (FC)≥2 representing positive log2FC (>1.0) and negative log2FC (<−1.0) were considered to be significantly different.

### Validation of DEMs by Real time quantitative PCR (RQ-PCR)

Eight miRNAs found to be differentially expressed in miRNA deep sequencing analysis were validated on CLL (*n* = 89) patients using locked nucleic acid (LNA)–based primers specific to each miRNA and SYBR green master mix as per the manufacturer’s instructions (Exiqon, South Korea) on Quantstudio 12 K Flex system (Thermofisher Scientific). The laboratory staff was kept unaware and blind to sample details. The data were normalized using geometric mean of two endogenous controls SNORD44 and SNORD48, and relative expression was evaluated using the comparative Ct method (2^−ΔΔCt^).

### Prediction of gene targets of DEMs and their functional pathways

The gene targets of all the eight significant DEMs were predicted by miRNet (http://www.miRnet.ca/). Hypergeometric test was applied to all the identified gene targets to evaluate the Kyoto encyclopedia of Genes and genomes (KEGG) functional pathways in miRNet.

### Gene expression profiling using microarrays

Total RNA obtained from PBMC of CLL patients (*n* = 21) and CD19^+^ sorted cells pooled from 10 healthy controls was labeled, amplified and hybridized on to G4851B Sureprint G3 microarrays (Agilent Technologies, Santa Clara, CA, USA), as described previously^26^. The gene expression data was log2 transformed and normalized using quantile normalization. The normalized data was analyzed by the Lima library from R-Bioconductor. Probes with an adjusted p-value less than 0.05 and log2FC of 1 were selected.

### Statistical analysis

The differences in gene expression obtained from RQ-PCR between CLL and healthy controls were compared using the Mann-Whitney Rank Sum test. Chi-square test was used to correlate the expression of DEMs with prognostic parameters. Time to first treatment (TTFT) was calculated from the date of diagnosis to the date of start of first therapy and overall survival (OS) was calculated from the date of diagnosis to date of last follow up or date of death due to any cause. For comparing cumulative incidence curves for risk of treatment, Gray’s test was used where death previous to any treatment was also considered as competing event. The log rank test was used to compare Kaplan Meier curves of OS. Variables having significant difference in univariate analysis were subsequently subjected to the multivariate analysis using Fine-Gray and Cox regression models for TTFT and OS respectively. Gray’s test and Fine-Gray modeling was performed using cmprsk library from CRAN R repository (https://cran.r-project.org/package=cmprsk) while rest of the statistical analysis was carried out with SigmaPlot V13.0 (Systat Software, Inc.).

### Data access

NGS and gene expression data generated in the study have been submitted to the NCBI Gene Expression Omnibus (GEO) (http://www.ncbi.nlm.nih. gov/geo/) under accession numbers GSE123436 and GSE81935 respectively.

## Results

### Identification of DEMs in CLL

**Fig. 2:**
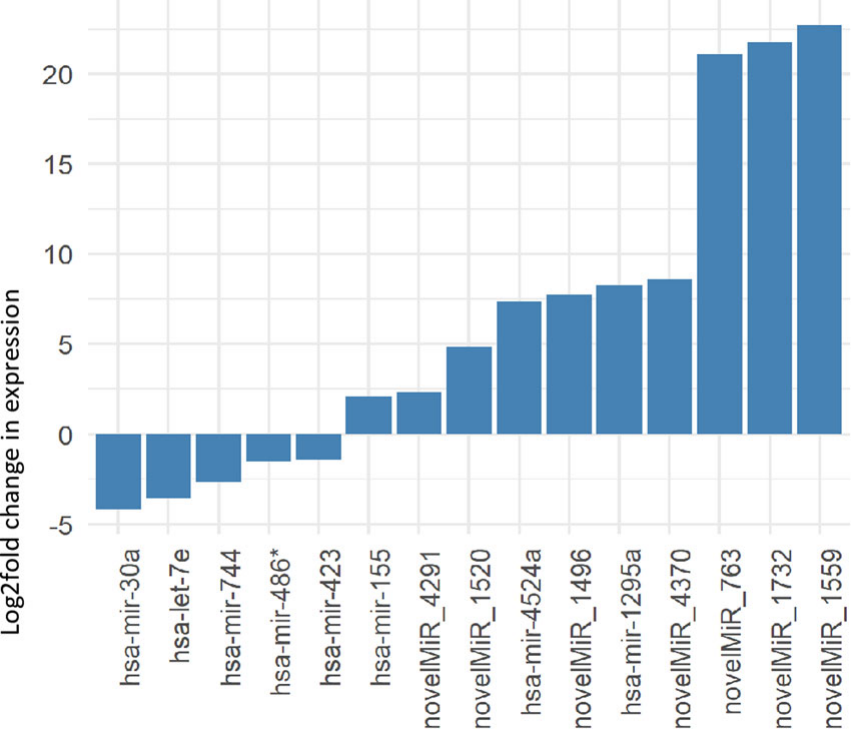
Histograms of relative fold changes of the eight differentially expressed miRNAs (DEM) as identified by RNA-seq.

A total of 239,039,053 raw reads were analyzed through the RNA-Seq pipeline which resulted in 872 miR sequences. Of these, fifteen sequences (8 known miRs: let-7e, miR-1295a, miR-155, miR-30a, miR-423, miR-4524a, miR-486, miR-744, and 7 novel miRs) were found to be differentially distributed and significantly deregulated in CLL (*p* adj ≤ 0.05; Fig. 2). Among the significant DEMs, miR-1295a (log2FC = 8.28), miR-4524a (log2FC = 7.39) and miR-155 (log2FC = 2.06) were up-regulated while miR-30a (log2FC = −4.19), let-7e (log2FC = −3.59), miR-744 (log2FC = −2.63), miR-486* (log2FC = −1.54), and miR-423 (log2FC = −1.41) were down-regulated in CLL.

### Annotation of novel miRs

The seven differentially expressed novel miRs identified with NGS (*p* adj. ≤ 0.05) were analyzed with DASHR and UCSC human genome browser for sequence annotations. Five of the novel miRs (novelmiR_4291, novelmiR_1520, novelmiR_1559, novelmiR_1732 and novelmiR_4370) showed homology with a multitude of tRNA molecules located on chromosomes 1, 6, 7, 9, 11, 12, 14, 15, 16, and 17 (supplementary table 1). The novelmiR_4370 showed homology with a piRNA-36225 (alias piRNA-28374; GenBank: DQ598159.1) as well. The novelmiR_763 got assigned on chr22 as piRNA-30799 (GenBank: DQ600599) and snoRNA U43 (SNORD43) (GenBank: X96642). Further characteristics of snoRNA were obtained from snoRNA Base (https://www-snorna.biotoul.fr/) and Rfam 14.1 (http://rfam.xfam.org/); and of piRNAs from piRNABase (http://www.regulatoryrna.org/database/piRNA/index.html) and isopiRNABank (https://mcg.ustc.edu.cn/bsc/isopiRNA/index.html). The mature miR sequence of novel miR_1496 did not yield any result in DASHR database but when aligned using BLAST at ncbi.nlm.nih.gov showed homology with a predicted uncharacterized LOC107984496 lncRNA at chromosome 12. When the precursor sequence of novel miR_1496 was analyzed by BLAST, it showed full alignment with 3′ end of tRNA-Ile (TAT)1–1 at Chr 19 (supplementary table 1).

### Prediction of DEM targets and networking of functional pathways

Eight significant DEMs identified by NGS were analyzed for putative gene targets in miRNet. A list of gene targets that were predicted for each of the 8 DEMs by miRNet is shown in supplementary table 2. These DEM targets consisted of several crucial driver genes of CLL such as ATM, TP53, NOTCH1(targets of miR-30a-5p), SF3B1 (target of miR-423-3p), and MYD88 (target of miR-155-5p). MirNet based network analysis of inter-miR interactions of 8 DEMs and with their targets suggested significant enrichment of various KEGG derived pathways (supplementary table 3) such as RNA transport (*p* < 0.001), pathways in cancer (*p* < 0.001), cell cycle (*p* < 0.001), mTOR signaling pathway (*p* < 0.001) and p53 signaling pathway (*p* < 0.001).

### Identification of differentially expressed genes (DEGs) by microarrays

**Fig. 3:**
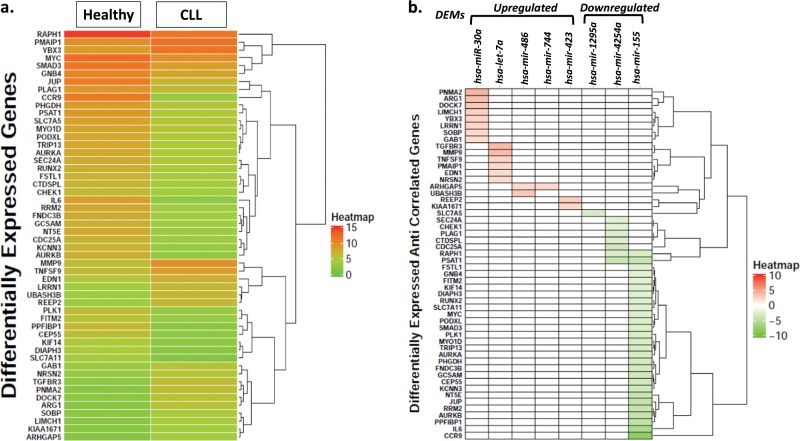
**a** Heat map of average expression values of 52 target genes of eight differentially expressed miRs (DEM) that were found to be deregulated using gene expression microarrays among CLL patients as compared to healthy controls. Each row represents average expression values of individual genes while the two columns represent average expression values of all the genes in healthy controls and CLL as indicated. **b** Anti-correlation of average expression values of 52 genes targeted by down-regulated or up-regulated miRs in CLL. Each column represents a DEM and each row represents a target gene.

Gene expression data revealed significant differential regulation of 736 genes of which 489 were up-regulated (*p* adj. = 0.05; log2FC ≥ 1) and 247 were down-regulated (*p* adj. = 0.05; log2FC ≤ 1) in CLL as compared to healthy controls. The intersection between the target genes of eight differentially expressed miRNAs and the differentially expressed genes revealed 94 genes (supplementary Table 4). Among these, 52 target genes displayed a distinct expression heatmap pattern in CLL as compared to healthy controls (Fig. 3a). Further, their expression values correlated inversely with those of DEMs as depicted in Fig. 3b. The down-regulation of target genes such as PNMA2, ARG1, TGFBR3 and others correlated with up-regulation of their corresponding regulatory miRs (miR-1295a, miR-4524a and miR-155). Similarly, the up-regulation of target genes e.g., CCR9, IL6, PSAT1 etc. correlated with down-regulation of their corresponding regulatory miRs (miR-30a, let-7e, miR-744, miR-486* and miR-423) (Fig. 3b).

### Validation of DEMs by RQ-PCR

Eight differentially expressed miRs identified by NGS were validated using RQ-PCR. As compared to healthy B-cells, miR-30a (FC = 0.06; *p* = 0.05), miR-423 (FC = 0.21; *p* = 0.034), miR-744 (FC = 0.03; *p* = 0.024), let-7e (FC = 0.14; *p* = 0.038) and mir-486 (FC = 0.166; *p* = 0.096) were down-regulated while miR-155 (FC = 6.39; *p* = 0.019), miR-1295 (FC = 74.2; *p* = 0.017) and miR-4524a (FC = 52.2; *p* = 0.026) were significantly up-regulated in CLL. These results are congruent to the pattern obtained from miRNA deep sequencing.

### Association of miRNA expression with prognostic factors and clinical outcome

In the validation cohort of 89 patients, IGHV mutation status, beta 2 microglobulin levels and genetic aberration data were available for 84, 87, and 84 patients respectively. The CLL-International prognostic index (IPI)^27^ could be calculated for 78 patients of which 13 were assigned as low risk, 31 as intermediate risk, 28 as high risk and 6 as very high risk patients. No significant association was observed for any of the DEM with IGHV status, beta 2 microglobulin levels and IPI score. The number of patients in each subgroup based on the genetic aberrations was too few to draw any statistical conclusion.

**Fig. 4:**
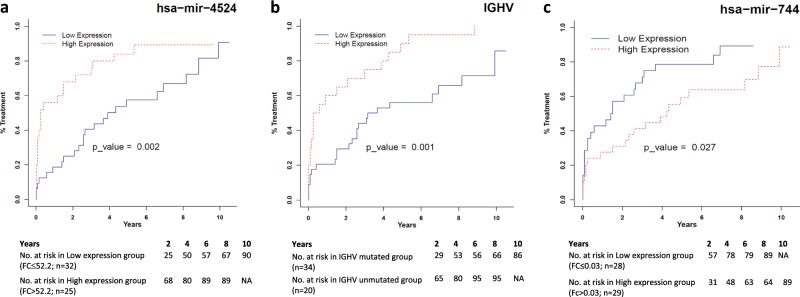
Cumulative incidence plots demonstrating risk of treatment in CLL patients stratified on the basis of level of expression of **a** miR-4524a, **b** IGHV mutation status, and **c** miR-744. The cut-offs for defining low and high expression of miRNA and the number of cases in each subgroup are shown below the curves. *p*-values and hazard ratios as obtained in the Fine- Gray model of multivariate analysis is shown inside the curve.

The expression level of differentially expressed miRs was further investigated for their association with TTFT and OS. The median fold change values of individual miRNAs measured by RQ-PCR were used as cut-offs to group the samples into high and low expression groups (Table 2). TTFT was compared in early stage CLL patients (Rai stage 0-II; *n* = 57) between the groups. The univariate analysis using Gray’s test identified miR-4524 (*p* = 0.002), miR-744 (*p*-0.027) and IGHV mutation status (p = 0.001) as significant parameters for evaluation of TTFT. Multivariate analysis with the significant parameters suggested that high expression of miR-4524a (HR: 1.916, 95% CI: 1.08–3.40, *p* = 0.026; Fig. 4a) and IGHV unmutated status (HR: 2.84; 95% CI = 1.58–5.120; *p* = 0.0005; Fig. 4b) were significantly associated with shorter time to first treatment while higher expression of miR-744 was found to be associated with longer TTFT (HR: 0.415; 95% CI = 0.224–0.769; *p* = 0.005; Fig. 4c). The OS was calculated for all the patients of validation cohort (*n* = 89). IGHV mutated patients displayed longer OS as compared to unmutated patients (*p* = 0.011). No association was observed between expression of any of the 8 DEMs with overall survival (Table 2).

**Table 2:** Association of differentially expressed miRNAs and other prognostic factors with time to first treatment (*n* = 54) and overall survival in CLL patients (*n* = 89).

Sr. No	Parameter (Cut-off)	Time to first treatment (TTFT)				Overall survival (OS)	
		Univariate	Multivariate			Univariate	Multivariate
		*p* Value	*p* Value	HR	95% CI	*p* Value	*p* Value
1.	hsa-let-7e (0.142)	0.897	−	−	−	0.25	−
2.	hsa-miR-30a (0.06)	0.437	−	−	−	0.66	−
3.	hsa-miR-155 (6.39)	0.272	−	−	−	0.058	−
4.	hsa-miR-423 (0.21)	0.471	−	−	−	0.647	−
5.	hsa-miR-486* (0.166)	0.748	−	−	−	0.647	−
6.	hsa-miR-744 (0.03)	0.0277	0.005	0.415	0.224–0.769	0.315	−
7.	hsa-miR-1295a (74.2)	0.127	−	−	−	0.971	−
8.	hsa-miR-4524a (52.2)	0.002	0.026	1.916	1.08–3.40	0.747	−
9.	β2 Microglobulin (3.5)	0.153	−	−	−	0.301	−
10.	Del(17p)	0.153	−	−	−	0.06	−
11.	IGHV status	0.001	0.0005	2.842	1.58–5.120	0.011	−

Gray’s test and log rank test was used to compare TTFT and OS respectively. Variables having significant difference in univariate analysis were subsequently subjected to the multivariate analysis using Fine–Gray model for TTFT

*NR* Not reached, *HR* Hazard ratio

## Discussion

The miRNA profiling in this study has identified differential regulation of eight important known miRs and seven novel sncRNA species related to tRNA, piRNA, and snoRNA that might contribute to the development/progression of CLL by targeting various crucial genes. Of the 8 known miRs, differential regulation of four miRNAs i.e., miR-155^7–9^, miR-486-5p^28^, miR-423^29^ and hsa-let-7e^29^ has been earlier reported in CLL. miR-30a which has been shown to have tumor suppressor role in lung cancer^30^ and breast cancer^31^ was found to be down-regulated in the present study. Of the various gene targets of miR-30a (Fig. 3), GAB1 which is a key molecule in the pathogenesis and progression of CLL was found to be up-regulated in the present study^32^. Higher mRNA levels of GAB1 have been shown to be associated with strong B-Cell receptor responsiveness and disease outcome in CLL^32^. Higher mRNA levels of GAB1 with low expression of miR-30 in the present study point towards a regulatory connection between these two which might be important for the malignant behavior of CLL cells. Like previous studies, the present study also reports up-regulation of oncogenic miR-155 in CLL^7–9^. Similar to previous reports, expression of miR-155 was not found to be associated with IGHV mutation status and survival outcome^7,33^. Unlike a previous report in CLL, down-regulation of miR-486 was observed in CLL patients in the present study^28^. Downregulation of miR-486 has been reported to contribute to the progression and metastasis of lung cancer due to increased expression of its target Rho GTPase activating protein 5 (ARHGAP5)^34^. In the present study, a concomitant downregulation of miR-486 and an upregulation of mRNA expression of ARHGAP5 suggests a pathogenic role of miR-486 in CLL. Perturbed levels of miR-1295 cluster located on chromosome 1 have been implicated in tumorigenesis in colorectal cancer and follicular lymphoma ^35,36^. As per a very recent report in CLL, miR-1295 was amongst one of the five most up-regulated miRs in CLL^37^. In the present study also, miR-1295 was the most abundantly expressed miR in CLL. Consistent up-regulation of miR-1295 suggests that it could be an important molecule in the pathobiology of CLL, however a detailed investigation for further functional characterization of its role in this process is required. Hsa-let-7e is yet another important miR that was downregulated in CLL in this study and has been implicated in several cancers. Similar studies on CLL such as by Filip et al.^29^ have also reported lower expression of let-7e in CLL as well as poor prognostic CD38^+^ CLL subgroup, which further supports observations of this study.

Incidentally, three of the eight DEMs observed in this study, namely miR-423, miR-4524a and miR-744 are located on chromosome 17. Levels of expression of miR-423, located at 17q11.2, have been shown to be influenced by an SNP rs6505162 C > A which has been shown to correlate with risk in a wide range of cancers although the mechanistic processes remain elusive^38^. In this study, expression levels of miR-423 were found to be reduced in CLL. A qRT-PCR based study has also reported reduced expression of miR-423 in CLL patients particularly in the context of higher lactate dehydrogenase (LDH) activity ^29^. Higher expression of miR-744 among older female patients with ovarian carcinoma has been reported to correlate with prolonged disease free survival, suggesting its protective influence^39^. This is comparable to findings in this study where higher expression of miR-744 correlates with extended time to first treatment in CLL patients as compared to those with reduced expression. The miR-4524a present at 17q24.2 is located within intron 22 of the host gene ABCA6. ABCA6 is an ATP binding cassette superfamily A, member 6 transporter which plays a role in macrophage lipid homeostasis. As per the present study, expression of miR-4524a was found to be upregulated in CLL, and a significant association was observed between its high expression levels and shorter TTFT. Furthermore, a study has shown upregulation of expression of ABCA6 in CLL^40^. It has been further shown that miR-4524a/b targets LDH A that promotes aerobic glycolysis in colorectal cancer and that it could become an important therapeutic target of cancer energy metabolism^41^.

Two novel miR sequences (novel miR-4370 and novel miR-763) having differential regulation in the present study, showed homology with two piRNA sequences (piR-36225 and piR-30799) and a C/D box snoRNA (U43 or SNORD43). Recent molecular studies have rediscovered the structural and functional diversity of snoRNAs^42^ and piRNAs^43^ and their aberrant expression in cancer. Induction of C/D box snoRNAs has been reported to favor leukemogenesis^44^. A number of snoRNAs have been reported to predict the clinical outcome in early stage as well as IGHV mutated CLL^45,46^. Various tRNA fragments have been reported to induce transient translational arrest^42^ and tRNA derived small RNAs can function similarly to miRs. Identification of tRNA molecules in the present study suggest that these might also be involved in the development of CLL.

**Fig. 5:**
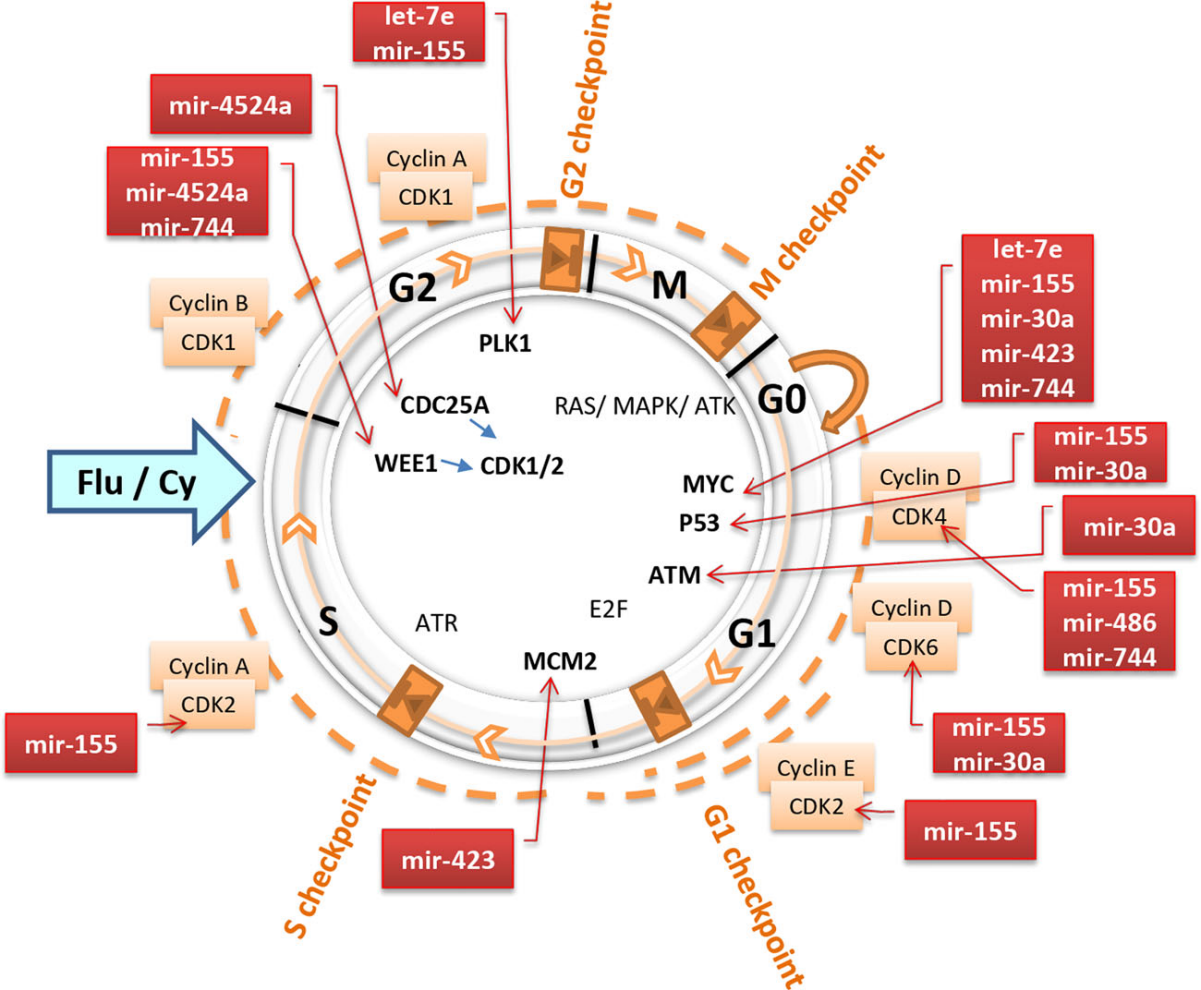
Putative functional roles of differentially expressed miRs (DEM) in regulation of cell cycle phases (M, G1, S, G2) and checkpoints (M, G1, S, G2) in CLL. The DEMs miR-155, miR-4524a, and miR-744 might disrupt CDK1/2 regulated G2 checkpoint by modulating the expression of CDC25A and WEE1. The let-7c and miR-155 may also act through PLK1 at the G2 checkpoint whereas miR-423 could abrogate expression of MCM2 at the S checkpoint. The let-7e, miR-30a, miR-423, miR-155, miR-744, and miR-486 are known to regulate TP53, MYC, and ATM and hence may modulate G1 phase of the cell cycle.

A thorough analysis of the targets affected by eight differentially expressed miRs suggested that these miRs may influence checkpoints of the cell cycle as depicted in Fig. 5. Information deduced from miRNet and published literature^47–50^ indicates that CDC25A and WEE1 that regulate CDK1/2 involved in G2 checkpoint may be abrogated by DEMs miR-155, miR-4524a and miR-744. Similarly, let-7e and miR-155 may also influence PLK1 at G2 checkpoint while miR-423 may modulate MCM2 at S checkpoint. Furthermore, MYC, P53 and ATM interactions during G1 phase may be affected by let-7e, miR-30a, miR-423, miR-155, miR-744, and miR486 (Fig. 5). It is plausible that these DEMs perturb intricate circuitry, result in altered apoptosis^51^ and cell arrest in CLL. Further investigations in miR(s) and target(s) are warranted to decipher mechanistic basis of oncogenesis in CLL and their effect on clinical outcome and response to therapy.

In conclusion, extensive studies aimed at a better elucidation of global transcriptional landscape of sncRNAs, their effects on clinical outcomes could help refine the patient stratification schemes and sncRNAs could surface as additional molecular biomarkers for improved prognosis and exploration of therapeutic targets in future.

## Supplementary information

Supplementary Data

## Data Availability

The NGS and gene expression data generated in the study have been submitted to the NCBI Gene Expression Omnibus (GEO) (http://www.ncbi.nlm.nih.gov/geo/) under accession numbers GSE123436 and GSE81935 respectively.
