# Supplementary material for: RNA-Seq profiling of deregulated miRs in CLL and their impact on clinical outcome
Source: Blood Cancer J. 2020 Jan 13;10(1):6. doi: 10.1038/s41408-019-0272-y (PMC6957689; doi:10.1038/s41408-019-0272-y)
Supplement: Supplementary file 1 — Supplementary Data [file 41408_2019_272_MOESM1_ESM.pdf]

Supplementary Table 1 : Genomic features of novel miRNAs differentially expressed in CLL. The sequences of these miRNAs were identified by miRDeep*; aligned with GRCh37, miRbase v22.1 and found to be novel. These unaligned sequences were annotated with DASHR that identified their genomic features as summarized in this table. The first column provides miR identities allocated by miRDeep*, followed by chromosomal localization (Chr), strand orientation (+ or -) (Strand), coordinates of chromosomal location of mature miRNA (Mature_loci), hairpin secondary structure of miRNA, sequence of mature miRNA (Mature miR), assignment of sequences by DASHR and their corresponding RNA sequences.

miR_ID identified by mirDeep*	Chr	Strand	Mature_ loci	HairPin secondary RNA Structure	Mature miR	Assignment by DASHR	Chromosomal location	RNA Sequence
Novel MiR_1732	Chr11	+	62126512-62126530	.....(((((((...((((((((..(((((((((....)))).)))))...).)))).))).....)))))))	atggttagcac tctggact	tRNA-Gln-CTG-1-4	chr15:66161400-66161471[-]	**gguuccaugguguaauggu**uuagcacucuggacucuga auccagcgauccgaguucaaaucucgguggaaccu
						tRNA-Gln-CTG-1-5	chr17:8023070-8023141[+]	**gguuccaugguguaauggu**uuagcacucuggacucuga auccagcgauccgaguucaaaucucgguggaaccu
						tRNA-Gln-TTG-1-1	chr17:47269890-47269961[+]	**ggucccaugguguaauggu**uuagcacucuggacuuuga auccagcgauccgaguucaaaucucggugggaccu
						tRNA-Gln-CTG-1-1	chr6:18836402-18836473[+]	**gguuccaugguguaauggu**uuagcacucuggacucuga auccagcgauccgaguucaaaucucgguggaaccu
						tRNA-Gln-TTG-3-1	chr6:26311424-26311495[-]	**ggccccaugguguaauggu**uuagcacucuggacuuuga auccagcgauccgaguucaaaucucggugggaccu
						tRNA-Gln-TTG-3-2	chr6:26311975-26312046[-]	**ggccccaugguguaauggu**uuagcacucuggacuuuga auccagcgauccgaguucaaaucucggugggaccu
						tRNA-Gln-CTG-5-1	chr6:27263212-27263283[+]	**gguuccaugguguaauggu**uuagcacucuggacucuga auccgguaauccgaguucaaaucucgguggaaccu
						tRNA-Gln-CTG-1-2	chr6:27487308-27487379[+]	**gguuccaugguguaauggu**uuagcacucuggacucuga auccagcgauccgaguucaaaucucgguggaaccu
						tRNA-Gln-CTG-2-1	chr6:27515531-27515602[-]	**gguuccaugguguaauggu**uuagcacucuggacucuga auccagcgauccgaguucaagucucgguggaaccu
						tRNA-Gln-TTG-3-3	chr6:27763640-27763711[-]	**ggccccaugguguaauggu**uuagcacucuggacuuuga auccagcgauccgaguucaaaucucggugggaccu
						tRNA-Gln-TTG-2-1	chr6:28557156-28557227[+]	**ggucccaugguguaauggu**uuagcacucuggacuuuga auccagcaauccgaguucgaaucucggugggaccu
						tRNA-Gln-CTG-1-3	chr6:28909378-28909449[-]	**gguuccaugguguaauggu**uuagcacucuggacucuga auccagcgauccgaguucaaaucucgguggaaccu
Novel MiR_4291	Chr 1	+	167683975-167683992	.(((((...(((((((((((((((((((...))).)))))..))))))))...)))))))))........	aggggtatga ttctcgct	tRNA-Pro-CGG-1-1	chr1:167683962- 67684033[+]	**ggcucguuggucuagggg**guaugauucucgcuucggg ugcgagaggucccggguucaaaucccggacgagccc
						tRNA-Pro-AGG-2-1	chr1:167684725-167684796[-	**ggcucguuggucuagggg**guaugauucucgcuuaggg ugcgagaggucccggguucaaaucccggacgagccc
						tRNA-Pro-AGG-2-4	chr11:75946557-75946628[+]	**ggcucguuggucuagggg**guaugauucucgcuuaggg ugcgagaggucccggguucaaaucccggacgagccc

						tRNA-Pro-AGG-2-5	chr14:21077495-21077566[-]	**ggcucguuggucuagggg**guaugauucucgcuuaggg ugcgagaggucccggguucaaaucccggacgagccc
						tRNA-Pro-AGG-2-6	chr14:21081560-21081631[-]	**ggcucguuggucuagggg**guaugauucucgcuuaggg ugcgagaggucccggguucaaaucccggacgagccc
						tRNA-Pro-TGG-3-2	chr14:21152175-21152246[+]	**ggcucguuggucuagggg**guaugauucucgcuuuggg ugcgagaggucccggguucaaaucccggacgagccc
						tRNA-Pro-TGG-3-3	chr16:3208923-3208994[+]	**ggcucguuggucuagggg**guaugauucucgcuuuggg ugcgagaggucccggguucaaaucccggacgagccc
						tRNA-Pro-CGG-1-2	chr16:3222049-3222120[+]	**ggcucguuggucuagggg**guaugauucucgcuucggg ugcgagaggucccggguucaaaucccggacgagccc
						tRNA-Pro-AGG-2-7	chr16:3232635-3232706[-]	**ggcucguuggucuagggg**guaugauucucgcuuaggg ugcgagaggucccggguucaaaucccggacgagccc
						tRNA-Pro-TGG-3-4	chr16:3234133-3234204[-]	**ggcucguuggucuagggg**guaugauucucgcuuuggg ugcgagaggucccggguucaaaucccggacgagccc
						tRNA-Pro-TGG-3-5	chr16:3238094-3238165[+]	**ggcucguuggucuagggg**guaugauucucgcuuuggg ugcgagaggucccggguucaaaucccggacgagccc
						tRNA-Pro-AGG-2-8	chr16:3239634-3239705[+]	**ggcucguuggucuagggg**guaugauucucgcuuaggg ugcgagaggucccggguucaaaucccggacgagccc
						tRNA-Pro-AGG-1-1	chr16:3241989-3242060[+]	**ggcucguuggucuagggg**guaugauucucgcuuagga ugcgagaggucccggguucaaaucccggacgagccc
						tRNA-Pro-CGG-1-3	chr17:8126151-8126222[-]	**ggcucguuggucuagggg**guaugauucucgcuucggg ugcgagaggucccggguucaaaucccggacgagccc
						tRNA-Pro-TGG-3-1	chr5:180615854-180615925[-]	**ggcucguuggucuagggg**guaugauucucgcuuuggg ugcgagaggucccggguucaaaucccggacgagccc
						tRNA-Pro-AGG-2-2	chr6:26555498-26555569[+]	**ggcucguuggucuagggg**guaugauucucgcuuaggg ugcgagaggucccggguucaaaucccggacgagccc
						tRNA-Pro-CGG-2-1	chr6:27059521-27059592[+]	**ggcucguuggucuagggg**guaugauucucgcuucggg ugugagaggucccggguucaaaucccggacgagccc
						tRNA-Pro-AGG-2-3	chr7:128423504-128423575[+]	**ggcucguuggucuagggg**guaugauucucgcuuaggg ugcgagaggucccggguucaaaucccggacgagccc
Novel MiR_763	Chr22	-	39715057-39715076	.....(((((.(.((((....(((((((((.((((...)))).))))...)))))).))))).)))))........	ctgattgtcac gttctgatt	piR-30799	chr22:39715057-39715084[-]	ACUGUGUG**CUGAUUGUCACGUUCUGAUU**
						snoRNA-U43	chr22:39715057-39715118[-]	CACAGAUGAUGAACUUAUUGACGGGCGGAC AGAAACUGUGUG**CUGAUUGUCACGUUCUG AUU**

Novel MiR_4370	Chr6	-	28641663-28641684	....(((((.((((((..(((((.(.(((...(..(((((...)))))..)...)))).)))))..))))))))))))..	ggggatgtagctcagtggtaga	tRNA-Ala-TGC-3-2	chr12:125406301-125406372[-]	**ggggauguagcucagugguaga**agcgcaugcuuugcauguaugaggccccggguucgauccccggcaucucca
						piR-36225	chr12:125406342-125406372[-]	**ggggauguagcucagugguaga**agcgcaugcu
						piR-36225	chr12:125424512-125424542[+]	**ggggauguagcucagugguaga**agcgcaugcu
						tRNA-Ala-TGC-4-1	chr12:125424512-125424583[+]	**ggggauguagcucagugguaga**agcgcaugcuuugcacguaugaggccccgguucaaucccccggcaucucca
						tRNA-Ala-CGC-3-1	chr2:157257281-157257352[+]	**ggggauguagcucagugguaga**agcgcgcgcuucgcaugugugaggucccgguucaaucccccggcaucucca
						piR-36225	chr5:180633868-180633898[+]	**ggggauguagcucagugguaga**agcgcaugcu
						tRNA-Ala-TGC-3-1	chr5:180633868-180633939[+]	**ggggauguagcucagugguaga**agcgcaugcuuugcauguaugaggccccggguucgauccccggcaucucca
						piR-36225	chr6:26553731-26553761[+]	**ggggauguagcucagugguaga**agcgcaugcu
						tRNA-Ala-CGC-1-1	chr6:26553731-26553802[+]	**ggggauguagcucagugguaga**agcgcaugcuucgcauguaugaggucccggguucgauccccggcaucucca
						piR-36225 at	chr6:28611222-28611252[+]	**ggggauguagcucagugguaga**agcgcaugcu
						tRNA-Ala-TGC-2-1	chr6:28611222-28611293[+]	**ggggauguagcucagugguaga**agcgcaugcuuugcauguaugaggucccggguucgauccccggcaucucca
						tRNA-Ala-AGC-4-1	chr6:28626014-28626085[-]	**ggggauguagcucagugguaga**agcgcaugcuuagcaugcaugaggucccggguucgaucccccagcaucucca
						piR-36225	chr6:28626055-28626085[-]	**ggggauguagcucagugguaga**agcgcaugcu
						tRNA-Ala-CGC-2-1	chr6:28641613-28641684[-]	**ggggauguagcucagugguaga**agcgcaugcuucgcauguaugaggccccggguucgauccccggcaucucca
						piR-36225	chr6:28641654-28641684[-]	**ggggauguagcucagugguaga**agcgcaugcu
						piR-36225	chr6_cox_hap2:133388-133418[+]	**ggggauguagcucagugguaga**agcgcaugcu
						piR-36225	chr6_cox_hap2:148225-148255[-]	**ggggauguagcucagugguaga**agcgcaugcu
						piR-36225	chr6_cox_hap2:163819-163849[-]	**ggggauguagcucagugguaga**agcgcaugcu
NovelMir_1496	Chr19	+	39902890-39902907	.......((.(((((((((((((((.(((....))))))))))....)))))))))).)).........	gccactggagctccggcc	lncRNA uncharacterized LOC 107984496	Chr12:41010206-41010221[-]	CCGGAGCTCCAGTGGC
						3' of tRNA-Ile (TAT) 1-1(TRI-TAT1)	Chr19:39412240-39412310[+]	AACCGATTGCGCCACTGGAGCTCCGGCCGCACCCCTGCGCCGGGGTCTTCTTCAGTGGTTCATAGCCACGT
Novel MiR_1520	Chr4	+	134189618-134189640	..(((.((((((..(((((......(..(((((......)))))..)....)))))..))	gcactctggactctgaatcca	tRNA-Gln-CTG-3-1	chr1:145963304-145963375[+]	**gguuccaugguguaauggugagc**cacucuggacucugaauccagcgauccgaguucgagucucgguggaaccu

				)))).)))............	gc	tRNA-Gln-CTG-3-2	chr1:147505038-147505109[+]	**gguuccaugguguaauggugagc**cacucuggacucugaauccagcgauccgaguucgagucucgguggaaccu
						tRNA-Gln-CTG-4-1	chr1:147737382-147737453[-]	**gguuccaugguguaaugguaagc**cacucuggacucugaauccagcgauccgaguucgagucucgguggaaccu
						tRNA-Gln-CTG-7-1	chr1:147800937-147801008[+]	**gguuccaugguguaaugguaagc**cacucuggacucugaauccagccaucugaguucgagucucugugggaaccu
						tRNA-Gln-CTG-4-2	chr1:149186125-149186196[+]	**gguuccaugguguaaugguaagc**cacucuggacucugaauccagcgauccgaguucgagucucgguggaaccu
						tRNA-Gln-CTG-1-4	chr15:66161400-66161471[-]	**gguuccaugguguaaugguuagc**cacucuggacucugaauccagcgauccgaguucaaaucucgguggaaccu
						tRNA-Gln-CTG-1-5	chr17:8023070-8023141[+]	**gguuccaugguguaaugguuagc**cacucuggacucugaauccagcgauccgaguucaaaucucgguggaaccu
						tRNA-Gln-CTG-1-1	chr6:18836402-18836473[+]	**gguuccaugguguaaugguuagc**cacucuggacucugaauccagcgauccgaguucaaaucucgguggaaccu
						tRNA-Gln-CTG-1-2	chr6:27487308-27487379[+]	**gguuccaugguguaaugguuagc**cacucuggacucugaauccagcgauccgaguucaaaucucgguggaaccu
						tRNA-Gln-CTG-2-1	chr6:27515531-27515602[-]	**gguuccaugguguaaugguuagc**cacucuggacucugaauccagcgauccgaguucaagucucgguggaaccu
						tRNA-Gln-CTG-6-1	chr6:27759135-27759206[-]	**ggccccaugguguaauggucagc**cacucuggacucugaauccagcgauccgaguucaaaucucggugggaccC
						tRNA-Gln-CTG-1-3	chr6:28909378-28909449[-]	**gguuccaugguguaaugguuagc**cacucuggacucugaauccagcgauccgaguucaaaucucgguggaaccu
NovelMiR_1559	Chr 15	-	45492643-45492721	....(((.((((((.(((...(((...(((((...(.((.....)).)...))).)).)))..))).)).)))).))).	cgtatagtggttagtactctgc	tRNA-His-GTG-1-1	chr1:145396881-145396952[-]	**gccgugaucguauagugguuag**guacucugcguuguggccgcagcaaccucgguucgaauccgagucacggca
						tRNA-His-GTG-1-2	chr1:146544773-146544844[+]	**gccgugaucguauagugguuag**guacucugcguuguggccgcagcaaccucgguucgaauccgagucacggca
						tRNA-His-GTG-1-3	chr1:147753471-147753542[+]	**gccgugaucguauagugguuag**guacucugcguuguggccgcagcaaccucgguucgaauccgagucacggca
						tRNA-His-GTG-1-4	chr1:147774845-147774916[-]	**gccgugaucguauagugguuag**guacucugcguuguggccgcagcaaccucgguucgaauccgagucacggca
						tRNA-His-GTG-2-1	chr1:149155828-149155899[-]	**gccaugaucguauagugguuag**guacucugcgcuguggccgcagcaaccucgguucgaauccgagucacggca
						tRNA-His-GTG-1-7	chr15:45490804-45490875[-]	**gccgugaucguauagugguuag**guacucugcguuguggccgcagcaaccucgguucgaauccgagucacggca
						tRNA-His-GTG-1-8	chr15:45492611-45492682[-]	**gccgugaucguauagugguuag**guacucugcguuguggccgcagcaaccucgguucgaauccgagucacggca

						tRNA-His-GTG-1-9	chr15:45493349-45493420[+]	**gccgugaucguauagugguuag**guacucugcguuguggccgcagcaaccucgguucgaauccgagucacggca
						tRNA-His-GTG-1-5	chr6:27125906-27125977[+]	**gccgugaucguauagugguuag**guacucugcguuguggccgcagcaaccucgguucgaauccgagucacggca
						tRNA-His-GTG-1-6	chr9:14433938-14434009[-]	**gccgugaucguauagugguuag**guacucugcguuguggccgcagcaaccucgguucgaauccgagucacggca

Supplementary Table 2 : Gene targets predicted for 8 differentially expressed miRNAs using mirNet

**Sr. No.**	**miRNA**	**Gene targets**
1	hsa-mir-744-5p	CYB5R3,TSC22D3,DUT,DVL1,CDC25B,CEBPA,CKB,CNN2,COL5A1,COPA,CTNNA1,CTNNB1,DDX11,FASN,FBN2,FGFR1,FKBP4,FOXO3,MTOR,FRZB,GAK,GART,GDI2,GFER,GMDS,GNB2,SFN,GSK3A,GSK3B,GSR,HIST1H1E,HIST1H2BD,HINT1,HMGA1,HNRNPC,HOXC11,HOXD11,HSPA1B,HSPD1,ILK,IRAK1,KPNB1,LAIR1,STMN1,PHC2,EEF1A2,EIF4A1,EIF4A2,EIF4G1,EN2,ENO1,F2R,FANCG,AARS,ABL1,ACTB,ACTG1,ACTN4,ADCY9,AP2A2,AP2B1,AKT2,NUDT2,ARHGAP5,ARHGDIA,ATP5B,ATP5G3,BCR,BRAF,CACNA1A,CAPNS1,CD44,VPS8,LARP1,SPECC1L,CTDNEP1,COTL1,NCS1,HAAO,SRRM2,WBP2,ZNF318,PRDX5,ULK3,CHD5,IRF2BP1,TSPAN17,AGO1,MRPL46,PABPC1,USP21,TRMT2A,PELP1,ARRDC2,ATP5S,DISC1,SERP1,EIF3K,DESI1,HTRA2,TOR2A,NOB1,CCDC106,EPN1,MINK1,MRPS16,TRAPPC12,MRPS2,DCXR,ABI3,IER5,FAM53C,MEX3C,FZR1,HPCAL4,UFC1,TMEM14C,MED15,PIAS4,TRIM33,CDK12,CHRAC1,MIEF1,FBXL19,ARL15,OTUD4,GATAD2A,NDE1,TOR4A,PXK,VPS37C,SAMD4B,LARP6,POMGNT1,TSR1,ATF7IP,ADAP2,FAM212B,LIN37,BARX1,WDR45B,SLC2A4RG,TM9SF3,UBQLN4,AGPAT3,CTNNBIP1,DDX24,PNPLA2,MRS2,PLEKHG5,GATAD2B,SCAF4,PRR12,SRGAP1,NUFIP2,NCEH1,JCAD,VPS18,NCKAP5L,PRX,CXCL16,UBL5,SENP2,UBE2O,RAB17,ANAPC1,FNDC3B,PLEKHG2,CCDC71,MRPL11,INTS3,C8orf33,ZSCAN18,LYNX1,DDX54,SECISBP2,PPDPF,PAGR1,NOL9,COLGALT1,FOXRED2,EDC3,PGGHG,FAAP100,MED28,KLHL15,SRCIN1,ILKAP,HM13,C6orf62,SH3BGRL3,FAM110A,BCL2L12,MARVELD1,L3MBTL2,RTL6,TMEM175,SLX4,LMNB2,SLC35B4,CGNL1,NKD1,TP53I13,ZNF598,DHX57,SLC25A46,MTSS1L,IGSF8,TJAP1,NACC1,SMYD4,OSBPL5,GPRASP2,PTPMT1,FOXP4,ART5,IRGQ,PPM1M,LDLRAD3,PTGR2,CDAN1,KCTD11,DUSP18,PPTC7,NOP9,PIKFYVE,USP12,ZCCHC24,CCNY,SGMS1,ANKRD52,INO80E,FAM171A2,TRIM59,ANKRD45,NANOS1,TMEM189,RNF207,HRNR,C1orf229,MEX3D,PIM3,RNASEK,ZNF704,WIPF3,FAM83G,LDLR,LGALS3,LIMS1,LIPA,LNPEP,LRP3,LY6E,MAN2C1,MELTF,MIF,MNT,ATP6,COX1,COX2,COX3,CYTB,MTHFD1,ND1,MYC,NCL,NDUFB10,NEUROD2,NFIX,NMT1,NOP2,NONO,NOTCH2,NRGN,YBX1,OPRD1,PA2G4,PAX2,CDK16,PDE4A,PDPK1,SLC25A3,PIGC,PIN1,PKP2,POLR2A,POLR2L,PPP1CA,PRKACA,MAPK7,PSMB2,PSMD4,PSMD11,PTMA,PTPRF,NECTIN1,ALDH18A1,PYGB,QARS,RAN,RHEB,RPL3,RPL4,RPL7A,RPL10,RPL13,RPL18,RPL18A,RPL37,RPLP1,RPS6,RPS14,SALL1,SAT1,SBF1,SFRP1,SRSF1,SH3GL1,SKI,SLC25A1,SMARCD1,SNAPC4,CAPN15,SREBF1,SRM,SURF4,SYPL1,TAPBP,TBX1,TBX2,TCOF1,TGFA,TGFB1,THBS2,TIAM1,TLE3,TSPAN7,TSC2,TUB,UBA1,

		UBE2A,UBE2N,VDAC1,ZNF155,TUBA1A,PRRC2A,BAG6,AIMP2,MAFK,ZNF212,MLF2,KMT2D,SYMPK,DPF1,NCOA3,ESS2,SMC1A,HIST2H2AA3,HIST1H2BC,PIP5K1A,ZNF282,IRS4,PDXK,KHSRP,STK16,TNKS,AP3D1,ARHGEF2,ZMYM3,RPS6KA5,CYTH2,DDX23,TECR,MICAL2,VGLL4,MATR3,IP6K1,URB1,AP5Z1,SEC16A,RBM8A,NUP153,NR1D2,FRAT1,FARSB,SCAMP2,G3BP1,TRIM28,ZNF256,SORBS3,STAM2,BET1,SF3A1,PAK4,TUBA1B,RACK1,C1D,TACC3,EIF3M,DDX17,CHERP,AGPAT2,POLD3,NFAT5,PTGES3,RAI1,GIPC1,TOB2,SRCAP,ERP29,TDRKH,ADRM1,OGFR,DIDO1,RASSF1,NXPH4,ATXN2L,LYPLA2,WDR37,CNOT1,UNC13A,SETD1B,TNRC6B,PHF8,GGA3,SLC35D1,ZCCHC14,PLEKHM2,TMEM131L,ATP11A,POFUT2,CLUH,MGRN1, ATMIN
2	hsa-let-7e-5p	FXN,GABPB1,GATM,NR6A1,GLO1,GLUL,EN2,ENSA,EPHA4,EZH2,FDPS,FMO4,FPR1,LYN,MXD1,MAGEA3,MAGEA6,MAGEA12,MDM4,MEF2D,MEIS3P1,MKI67,MMP9,MPL,ATP6,COX1,COX3,ND1,ND2,ND3,ND4,ND5,MYC,GNG5,GOLGA4,GPM6B,GTF2I,GTF3C1,HIST1H2BD,HLAC,HMGB1,HMGA1,HNRNPC,NDST1,IGF1,IGF1R,IL6R,CXCL8,ITGA3,IVD,KPNA5,RPSA,LMNA,ACTA1,ADH5,AHR,AK4,AMD1,AMPD2,XIAP,FASLG,AQP6,ARCN1,BACH1,CCND1,POLR3D,CALU,RUNX1T1,CCNG1,CCNT2,CD59,CDC5L,CDH18,CDKN1A,AP1S1,CLTC,COL6A1,COL8A1,COX6B1,COX10,CLDN4,CREBBP,CRK,CRX,CRY2,CTPS1,CYP2B6,DHX15,DIAPH1,DNA2,DNAH9,ARID3A,DSP,DTNB,DUSP1,DVL3,E2F6,EDN1,EIF4A1,EIF4EBP2,EIF4G2,TGOLN2,ARID3B,LEFTY1,IGF2BP1,IGF2BP3,CELF1,CELF2,SLC12A7,RAI2,WDR4,ZNF460,PGRMC1,SUB1,YWHAQ,HNRNPUL1,RABL2B,RABL2A,SEC23IP,CA5B,ATXN2L,IKZF3,COPG1,ZNF652,RNF44,FNDC3A,DZIP1,SCMH1,PDCD11,WDFY3,DAAM1,ZNF609,PEG10,MCF2L2,PLXND1,GGA3,TTLL12,PSME4,PMPCA,PACS2,RPRD2,ICOSLG,KIAA0930,PSD3,AHCYL2,MED13L,TDRD7,CBX5,PHF3,PES1,MACF1,SUZ12,RBFOX2,PIGN,ACOT9,PLD3,MTCH2,BRI3,NOCT,ARIH1,INTS7,ZNF451,APPL1,CHTOP,SERBP1,RSL1D1,FBXW2,AGO1,AGO2,SALL3,DISC1,POLL,SGSM3,SLCO4A1,SLCO3A1,C19orf53,CCDC113,GTPBP8,THYN1,RACGAP1,ABT1,CCDC106,ERO1A,SOCS7,DCAF8,SHANK1,TMED5,MRPS2,NAA20,RNFT1,PLEKHO1,CPA4,PIGP,COX16,C1RL,THEM6,RWDD1,CWC15,NCKIPSD,TRMO,LSR,SLC38A2,MIEF1,DGCR8,FAM105A,UHRF1BP1,CDKAL1,SEMA4C,DNAJC28,PARP16,ZNF770,USP47,BSDC1,FIGN,VPS13D,ARL8B,C11orf57,LRRC20,SLC38A7,NUDT15,TBC1D19,SYNJ2BP,PLCXD1,YOD1,CDV3,KIF27,BMP2K,OTUD5,OTUB1,KRBOX4,CHD7,HIF1AN,RABL6,IPO9,FAM222B,RCOR3,TXLNG,DCAF6,USE1,ECHDC1,ZKSCAN7,LRRC8A,DIABLO,SAR1A,RAD18,UGGT1,STARD7,SMARCAD1,NCLN,ATXN7L3,YAE1D1,C12orf4,FAM219B,SCYL1,RHBDD2,KIAA1143,MTUS1,KIAA1328,PDP2,BAHCC1,WDR48,USP37,FAM160B1,NCKAP5L,IGDCC4,RAB40C,ABHD17C,SPCS3,THADA,PRSS22,RRAGC,NSD1,MTMR14,NOM1,SMAP2,MYCN,MYO9B,NAP1L1,NCBP1,NDUFA3,NDUFS5,NF1,NME4,SLC11A2,NUCB2,OPRL1,PA2G4,PAFAH2,PAX3,PBX2,PCBP2,PDGFB,GATB,PFAS,PGD,PHKA1,PLAGL2,PLCG2,PLK1,PMAIP1,POLD1,POLR2D,CTSA,PPP1R2,PPP1R10,PPP2R1A,PPP2R2A,PRIM2,MAPK6,MAP2K7,PSMA6,PSMD2,PTK2,QSOX1,PYCR1,QDPR,RANBP2,RAP1A,RBBP4,RBM4,RDX,RFC2,RHD,RPA1,RPL10,RPL12,RPL27A,RPLP2,MRPL12,RPN2,RPS27,RRAD,RRM1,RRM2,SALL1,MSMO1,ATXN2,SCD,SRSF2,SKIV2L,SLC12A4,SLC20A1,SOD2,SPN,SPTBN1,SQLE,SREBF1,SSB,STAT2,STAT3,STK4,STRN,STX3,SUOX,SUPT4H1,SURF4,SVIL,VAMP2,SYT1,TCF4,TERF1,TGFBR3,THBS1,TIMP3,TRAPPC10,TNFAIP1,TNFAIP3,TNFRSF1A,TUBB2A,TXNRD1,UBE2H,UBE2V2,SUMO1,VARS,VCL,TRPV1,WNT1,YWHAE,YWHAG,YWHAZ,ZNF8,CNBP,ZNF28,ZNF200,ZFAND5,ZNF236,LUZP1,PRRC2A,STAM,MLLT10,KMT2D,HMGA2,COIL,NCOA3,SMC1A,ARID1A,TRRAP,FZD9,DYRK3,DYRK2,IRS4,SOCS1,IRS2,EIF3J,RNMT,TNFSF9,TNFRSF10B,PEX11B,GMPS,SLC5A6,MBD2,BTRC,WASL,BAZ1B,SPAG9,CLDN12,ATG12,AURKB,TIAF1,NOLC1,ATP6V1F,PPIG,HAND1,ZNF264,ONECUT2,ATP6V1G1,NUP155,MTFR1,CEP135,VGLL4,BZW1,KIAA0391,ESPL1,KIAA0100,HERPUD1,KIAA0355,SETD1A,EIF4A3,MATR3,SNX17,SPCS2,TSC22D2,AREL1,UBAP2L,ARNT2,ZBTB5,RBM8A,SUGP2,SERF2,ZNF256,LHFPL2,ZNF443,AKAP8,KA

		TNB1,PCGF3,TUBA1B,TUBB4A,TUBB4B,RBM14,LRRC41,CARM1,SEMA4B,PRPF8,PDLIM5,MARS2,SDR42E1,ZFAND4,TBC1D31,PIGS,PDZD8,LRIG3,AEBP2,NAA30,MSI2,ZFP3,IRGQ,C19orf47,TRIM71,DCBLD2,FAM43A,UROC1,GRPEL2,PM20D2,UBXN2B,GPAT4,BRI3BP,SMCR8,ZNF578,ZNF417,ZNF738,C1orf210,DTX3L,CEP120,ANKRD46,TMTC3,OR7D2,IFNLR1,PAPD4,TXLNA,KLHDC8B,ZNF584,PDE12,HIPK1,SLC16A9,JMJD1C,SPATA13,JAZF1,TMED4,UBN2,NUDT8,RNF144B,MFSD8,PGM2L1,KCTD21,C5orf51,ZBTB8OS,NAT8L,ACER2,RMND5A,NT5DC2,MARCKSL1,PLEKHA3,PHACTR4,SLC2A11,ATG9A,RNF26,EFHD2,ADIPOR2,RHBDF2,LIN28A,C12orf49,AGMAT,ALG13,NAA60,NRSN2,ZNF556,CTC1,CHD9,OPA3,EDEM3,SLC19A3,LIMD2,COLEC12,RCC1L,C1orf21,ZNF611,CDCA3,DNAL1,HASPIN,KREMEN1,EMILIN2,KATNAL1,SLC10A7,TOMM40L,UTP15,ZNF644,NOA1,NICN1,TMEM107,RAB11FIP4,ZBTB37,USP38,FUT10,C19orf48,ZNF587,PPP1R15B,FAM104A,ZNF566,FBXL20,ARHGAP19,COX14,HIST1H2BK,ZCCHC3,LMLN,MIDN,HS6ST2,CCDC97,ZNF799,DHX57,FMNL3,ANKRD40,ELMSAN1,CABLES1,ZC3HAV1L,TIMM50,RAB19,MS4A10,ZNF774,ZNF284,MTX3,SKA2,NDUFA4P1,NHLRC2,NHLRC3,BEND4,RBM12B,ZNF805,PRAMEF13,OCLN,CENPP,POTEG,FNDC9,HIST2H2BF,ATXN7L3B,PRR5,ARHGAP8,POTEM,ZFP62,FAM83G,FOXD4L6,CASTOR2,C11orf91
3	hsa-mir-1295a	ACTB,GJB2,NDUFA7,SH3BGRL,BSND,MFAP5,SLC7A5,ARL4C,CD3EAP,PGRMC1,BVES,PABPC1,MOB1A,TWNK,CASP16P,NOL9,DSN1,DDI2,MAP3K21,NT5C1A,ZNF607,ZBTB45,TEX261,TRAPPC6B,FAM241A,NUDCD2,AGO3,ZBTB8B,EPOP,OCLN
4	hsa-mir-155-5p	AGTR1,ASPH,ATP6V1C1,AXL,BACH1,AKT1,ALDH9A1,ALDH3A2,ANPEP,ANXA2,APAF1,APC,RHOA,ASNS,CDK5,CDKN1B,CDKN2A,CEBPB,CFL2,CLTA,CLTC,COL4A2,CPD,CPT1A,CRAT,CS,MAPK14,CSE1L,CSF1R,CSNK1A1,CSNK1G2,CSRP2,BCAT1,CCND1,BCL6,KLF9,CALU,CARS,CASP3,CAT,RUNX2,CBFB,CBL,CCND2,CCNT2,CD36,ENTPD1,CD68,CD81,CDH2,CDH6,CDH13,CDK2,CDK4,DNAJB1,ICAM1,IFNGR1,IGF2R,CYR61,JCHAIN,RBPJ,IL2,IL6,CXCL8,IL13RA1,INPP5A,INPP5D,EIF3E,ITGB4,ITGB5,ITK,JARID2,JUN,JUNB,JUP,KCNN3,KIF22,KPNA5,TNPO1,KRAS,KRT6B,FADS1,LPL,TACSTD2,MARCKS,SMAD1,SMAD2,SMAD3,SMAD4,SMAD5,MCAM,MECP2,MEF2A,MEIS1,MEST,MIA2,MGST2,MITF,MKLN1,MLH1,MAP3K10,MMP16,MOV10,MPP2,MSH2,MTAP,MUT,MXI1,MYB,MYBL1,MYC,MYD88,MYLK,CTLA4,CTNNA1,CTNNB1,CUX1,CYP1A1,CYP51A1,DAG1,DBN1,ECI1,DDB2,DDX10,TIMM8A,DHCR24,DMD,DNMT1,DOCK1,DR1,DSG2,E2F2,S1PR1,EDN1,PHC2,EEF1A2,EEF2,EGFR,EIF4A1,EIF4G2,ELK4,EPB41L2,EPRS,ESRRA,ETS1,EZH1,F5,FDFT1,FGF2,FGF7,FKBP3,FOXE1,FOXO3,FLI1,FLNA,FLNB,FLT1,FOS,GALC,GATM,B4GALT1,GLB1,GCLC,GLG1,GNAS,GPM6B,NR3C1,GSK3B,MSH6,HAL,HIF1A,HIVEP2,HK2,HLADPA1,HMGCS1,ACOX1,ADAM10,ADD3,ADH5,AP1G1,AGL,SGPL1,FUBP1,EIF2B2,EIF2B5,BUD31,FUBP3,MPZL1,MAP3K14,SOCS3,UBA3,DOK2,CLDN1,USP8,INA,CNOT9,AIFM1,SLC33A1,AURKB,XPR1,NOLC1,AIMP1,BCL7C,SOCS6,TRIP13,VAMP3,SNAP29,SLC9A3R2,CIAO1,EIF4E2,ADAMTS4,EEF1E1,SCAMP1,VPS4B,BAG5,ZNF254,H2AFY,CDC42BPB,NUP155,FEZ2,IKBKE,SH3PXD2A,TTC37,MARF1,PHF14,N4BP1,CLINT1,RAPGEF2,DOCK4,PHACTR2,MATR3,SERTAD2,CKAP5,TOMM20,NCAPD2,LPGAT1,KIF14,MAFB,DMTF1,CHAF1A,UBA2,NR1H3,ARPC3,ACTR2,TSPAN3,NAMPT,HNRNPA3P1,ABI2,PSME3,PATJ,DCAF7,ABCC4,BET1,RTN3,NSA2,PGRMC2,SEC24B,FAM3C,HAX1,ZBTB18,DDX17,IPO8,SSSCA1,P3H3,ARL6IP5,SLC35A1,CCT2,DRAP1,PDLIM5,RRAGA,GNA13,NFAT5,STAG2,NES,NCKAP1,ZNF273,MTHFD2,CCR9,CD3EAP,FGL2,MAN1A2,PAPOLA,SPIN1,EHD1,PRSS21,TOMM34,METAP2,SLC27A2,GLIPR1,NUPL2,KIF3A,CDC37,FSTL1,PSIP1,AKAP10,PRAF2,PDCD10,PACSIN2,ACOT7,PDAP1,EXOC3,COG2,HSPA4L,ZNF652,RAB11FIP2,AAK1,INPP5F,TRAK1,SACM1L,SHANK2,TRIM32,PALLD,KDM1A,CLUAP1,MRPS27,TAB2,PLXND1,RGL1,RCOR1,CYFIP1,GANAB,FAM120A,PSME4,SYNE2,ANKRD12,CAMTA1,EXOC7,WWC1,NEMP1,DPY19L1,UBR4,UFL1,KIAA0368,EXOSC2,SIRT1,TRAM1,SUZ12,MORC3,SLC39A14,SKIV2L2,CARHSP1,LEMD3,NUP62,LDOC1,ARFIP2,SLC7A11,ZKSCAN5,SH3BP4,GABARAPL1,IFI

		T5,ATXN10,METTL7A,INTS7,CNOT10,GEMIN5,CLIC4,MYO1D,MYO1E,MYO6,MYO10,NARS,NASP,NEU1,NEUROG1,NFKB1,NFYC,NKX31,NOS3,NOTCH2,NOVA1,SLC11A2,NT5E,OLR1,OXCT1,PEBP1,PAK2,PCCA,PCCB,PCDH9,PCNT,PDE3A,PDK1,PFDN4,PIK3CA,PIK3R1,PLAUR,PLK1,PLS1,UBL3,PODXL,POLR2C,CTSA,PPL,PPM1G,PPP2R2A,PPP5C,PKIA,PRKAR1A,PRKAR2A,PRKCI,PKN2,MAPK13,HTRA1,PSEN1,PTEN,TWF1,PTMS,PTN,PTPRJ,RAD1,PYGL,RAB2A,RAB3B,RAB6A,RAB27B,RAB5C,RAC1,RAD23B,RAD51,RAP1B,RARS,RCN2,RHEBP1,RHEB,RING1,RNF2,RORA,RP2,RPL39,RPS20,RREB1,RRM2,S100A11,SCD,CCL2,SDCBP,SEL1L,SELE,SRSF1,SRSF2,SKI,SLC1A5,SLC7A1,SLC12A4,SMARCA4,SMARCD2,SMARCE1,SNTB2,SP1,UAP1,SPI1,SRPK2,TROVE2,STAT1,STAT3,STIM1,AURKA,STRN,STX5,STXBP2,SUPT5H,SYPL1,TBCA,TCEA1,TCF4,GCFC2,TCF12,TMBIM6,TERF1,TFAM,TFCP2,TFPI,TGM2,THBS1,THRB,TJP1,TLE4,TNFAIP2,TPBG,TPD52,TPP2,TRIO,TRPS1,TTF1,TXNRD1,TYRP1,UBE2D2,UBE2D3,UBE2G1,UBE2H,UGDH,UGT8,UQCRB,UQCRFS1,VAV2,VBP1,VCAM1,VHL,LAT2,WEE1,WNT5A,WRB,XPC,XPNPEP1,XPO1,YWHAZ,ZFP36,ZIC3,ZNF28,ZKSCAN1,ZNF148,VEZF1,ZNF207,ZNF236,SLC30A1,LUZP1,DEK,ALDH5A1,ABHD16A,CHAF1B,PNPLA4,PICALM,STK24,CUL4B,PPFIBP1,YBX3,COPS3,DEGS1,YARS,AKR7A2,PSMG1,AKR1C3,SOCS1,DDX3Y,EIF3A,EIF3C,EIF3F,EIF3G,EIF3J,PEA15,FADD,TNFRSF10A,TRIM24,GMPS,ALDH1A2,FNDC3B,GNPNAT1,RAPH1,WNK1,DDRGK1,MRPS34,SECISBP2,KDELC1,RBM42,DCAF10,CDC73,CARS2,NAA16,DYNC2H1,DHX40,FASTKD1,SAP30L,COLGALT1,CCDC82,L2HGDH,ERMP1,JADE1,NAA25,VCPIP1,ZNF703,THOC7,MUS81,CHD9,NAA50,EDEM3,GRPEL1,CDK5RAP3,CPEB4,WDR82,LNPK,FIP1L1,CAB39L,TSPAN14,DIAPH3,VANGL1,ZNF611,HSDL1,MARVELD1,LONP2,RAB34,FCAMR,OBSCN,ARMC2,RAB6C,ZNF644,ANTXR1,POLR1B,FAR1,PHF6,CMSS1,CARD11,MCM8,KIAA1841,GPT2,CBR4,TBRG1,NFATC2IP,MASTL,TNKS1BP1,SSH2,PNPT1,MIDN,OTULIN,ZNF468,ZNF160,ERI1,FMNL3,CHURC1,CHRDL1,ZNF300,INTS4,OXNAD1,UBTD2,SPECC1,SLC38A5,ZNF561,TADA2B,PAXBP1,TP53INP1,CEP41,RDH13,CYP2U1,FMNL2,OSBPL9,OSBPL10,TMEM123,RAB3IP,SSX2IP,UBE2J2,ANAPC16,IKBIP,CSNK1A1L,TTC8,MTFMT,MSI2,OVCA2,SWSAP1,FITM2,DNAJC19,PRRC1,CD109,UBXN2B,FAM199X,ASB6,BRI3BP,KDELC2,KRT80,ZNF98,ZNF714,SLC30A7,WTH3DI,GLIPR2,CREBRF,TMEM167A,MBLAC2,FAM91A1,MOSPD2,TMTC3,SPRED1,DENND1B,ZNF431,ZNF384,AGO4,ARID2,FAM76A,PDE12,TAPT1,C3orf58,TYSND1,TMEM136,HNRNPA3,ARL5B,TDRD6,FOXK1,LNX2,RICTOR,ZBTB38,LCLAT1,MMS22L,LCORL,GCSAM,FAM177A1,FAM98B,KANSL1,ZNF493,EOGT,ARL10,PAXX,CCDC137,ZNF260,ZNF678,AMIGO2,GEN1,TICAM2,IRF2BP2,PTAR1,AGRN,KMT5A,CISD2,EIF3CL,MTRNR2L7,MTRNR2L1,MTRNR2L3,MTRNR2L5,MTRNR2L9,DCUN1D2,ETNK2,OGFOD1,FBXW7,LIN7C,SAMHD1,SIN3A,KBTBD2,KANK2,RPAP1,UPF2,LTN1,ZNF500,RAI14,HERC4,CHTOP,GAPVD1,ARL5A,PHGDH,SERGEF,CNNM3,INTS6,TIMM13,HBP1,DNAJC2,CNPPD1,GHITM,TAF5L,PALD1,BRPF3,ARFIP1,PDCD4,LSM3,RAB30,MAT2B,HDHD5,OSTM1,SPCS1,NOB1,MRPL18,SSU72,PACSIN3,TMOD3,GMPPA,DPP7,STRN3,PSAT1,UBQLN2,UBQLN1,PNPLA8,TPRKB,PAM16,GOLT1B,VPS36,UBXN1,TXNDC12,NMD3,KLHL5,MECR,IER3IP1,KCTD3,CEP83,HSD17B12,C3orf18,KRCC1,MEX3C,CDC40,PCYOX1,REV1,UBE2J1,HSD17B7,ANKFY1,RTFDC1,LARS,RAB14,CUTA,ATP6V1H,LUC7L2,FAM96B,HSPB11,SARAF,SELENOT,RAB23,CAB39,LUC7L3,RSF1,BTBD1,TM6SF1,POLE3,CHRAC1,GAR1,KCTD5,PLEKHA5,TMX3,PUS7,GNL3L,ARL15,SLC35F2,LY6K,KLHL28,QPCTL,WBP1L,QRICH1,RETSAT,TBC1D8B,NSD3,DNAAF5,IMPAD1,MRPL16,,C17orf80,PDPR,DET1,ARGLU1,TMEM33,CEP55,INTS10,RIF1,PBRM1,RPRD1A,ARL8B,ERGIC1,MRS2,CNOT6,MAVS,RPTOR,STRBP,LRRC59,IL17RB,SOX6,CDV3,TRMT1,LRRC40,CHD7,INTS8,YEATS2,NSUN5,RBM22,WDR11,CNDP2,,ZNF83,EXOC2,RIOK2,LRIF1,MBNL3,KDM3A,ERBIN,SLC25A40,CTNNBL1,POLE4,MFF,CIAPIN1,C16orf62,TWSG1,AGTRAP,ATP13A1,PELI1,CORO1B,SLC39A10,ZNF248,GPAM,CHD8,ZSWIM6,RBAK,SNX6,TBC1D14,KLHL42,FAM135A,WDFY1,HOMEZ,ZNF492,TSHZ3,VPS18,

		MPP5,HHIP,ATG3,NUCKS1,ATPAF1,PLEKHA2,GNB4,C12orf10,SLC25A19,RNF123,GOLPH3,NCAPG,ATL2,1-Mar,CREB3L2
5	hsa-mir-30a-5p	ARG1,ATF1,ATM,ATOX1,ATP2A2,ATP6V1B2,ATP6V1C1,ATRX,BAX,BCL2L1,BCL9,RHOB,RND3,SERPINC1,CDC20,CDH1,CDH2,CHD1,CIRBP,TBCB,AP2S1,AP3S1,CNP,CPOX,CREM,CSNK1G2,CSNK1G3,CTNNB1,CTNND1,CTSL,DAG1,DHX8,DDX10,TIMM8A,DNMT1,ARID3A,DSG2,BDNF,PRDM1,BNIP3L,FOXL2,ZFP36L1,KLF9,CAPN2,CAST,CARS,CASP3,CAT,RUNX2,CBFB,CCNF,ENTPD1,CD44,JUP,KCNN4,KIF5B,KIF11,KPNA1,KPNA4,KRT6B,KRT85,STMN1,LCP1,LDLR,LIFR,LIG3,LOX,LSS,SMAD1,MAN2B1,MAT2A,MBNL1,MET,MFAP3,MGST2,CD99,MLH1,MPP2,MRE11,MTR,MYBL2,MYO10,PPP1R12A,HNRNPM,NAP1L1,NCAM1,NCL,NDUFV3,NEUROD1,NOTCH1,NPR3,NT5E,NUCB1,OGDH,OPHN1,PAFAH1B2,SERPINE1,PAWR,PCNA,PCNT,PGGT1B,PGM1,PGM3,SERPINB5,SERPINE2,PIK3C2B,PIK3CD,PIK3R2,PKM,PLA2G4A,PLAGL2,PLSCR1,PLXNA1,EXOSC10,UBL3,POLR2B,POLR2C,PPA1,EEF1A1,EEF2,CELSR3,EGFR,EIF2B1,EPB41,NR2F6,ERG,ESR2,ESRRA,EYA2,ACSL4,FANCF,FBN1,FDX1,FOXG1,FOXD1,FRG1,FUCA1,GAB1,GALNT1,B4GALT1,GCLC,GLRX,GMFB,GNAI2,GNG5,GOLGA1,GPD2,GTF2E2,GTF2H1,GTF2H2,HIST1H1A,HDAC1,HDGF,HK1,FOXA1,HNF4G,HNRNPA1,PRMT1,DNAJA1,HSPA5,DNAJB1,FOXN2,IDH1,IFNAR2,IFRD1,IGF1R,IL1A,ITGA6,IREB2,ITGA2,ITGB3,ITGB4,JAK1,JUN,ABL1,ACP2,ACTC1,PLIN2,AP2A1,AK2,ANPEP,ANXA1,APEH,APLP2,TRIM23,ARF6,NFAT5,TRAF3IP2,ARPP19,NCKAP1,ZNF460,RPP40,SEC24A,FRS2,ME3,RAB10,CPSF4,MAN1A2,DBF4,TMED2,MLLT11,TMED10,ASCC3,GLIPR1,TMED1,DNAJB4,RPP14,PDCD10,XPOT,PHB2,CBX3,GABARAPL2,IKZF2,MTF2,VASH1,ZNF507,ANKRD26,ENPP4,MLXIP,BAHD1,HABP4,LIMCH1,KLHDC10,RAB21,RBM34,MAST3,XPO7,ERP44,VWA8,AVL9,PEG10,HIC2,CLCC1,LARP4B,UBXN4,FAF2,PSME4,NEDD4L,DPY19L1,JADE2,SMCHD1,LARP1,TMED3,SF3B3,ABCB10,SEC11A,PES1,LRRC8B,TNPO3,RBFOX2,DDAH1,CORO1C,CD2AP,ZMYND8,KPNA6,SLC7A11,STX12,RAB38,RASGRP3,ZNF324,ATXN10,HECTD1,SAMHD1,TMEM87A,TBC1D10B,GLCE,LTN1,SACS,INTS6,TIMM10,TIMM9,PABPC1,DNAJC2,VPS41,AFF4,AGO2,NFU1,LSM1,RBMS3,TNRC6A,ZNF638,PRPF19,EML4,DROSHA,NXT1,MYLIP,UBN1,CPSF1,PYCR2,NRBP1,IL21R,TMED5,TMED7,RRP15,NDUFAF1,UTP11,ASB3,DYNC1LI1,DCTN4,NT5C3A,IER5,CHST15,NIP7,ANAPC5,FAM8A1,RNF138,DTL,RAB14,UFM1,AZIN1,CPSF3,ATP8A2,SIX4,GALNT7,BCL11A,BTBD1,CSNK1G1,DCUN1D1,GNG2,SLC38A2,KCTD5,TMCO1,RHOF,WDR74,,RBFOX1,OTUD4,SAMD9,NDE1,PGPEP1,RETSAT,UHRF1BP1,DNAAF5,INTS11,PAK1IP1,NOL8,SOBP,FANCL,LRRC8D,DARS2,PNPO,MSL2,SEC61A2,RNF220,RIF1,PBRM1,RPRD1A,SETD5,SLC38A7,OGFOD1,PCMTD2,C7orf43,LIN7C,SLC35C1,DNAJA4,MED29,THUMPD1,PRPF40A,CDC37L1,LMBR1L,POLR3E,PPID,PPOX,PPP1CC,PPP1R2,PPP1R10,PPP2R1B,PPP2R2A,PTPA,PPP2R5C,PPP3CA,PPP3CB,PPP3R1,PRIM1,PRKAA1,PRKAR1A,MAPK1,MAPK8,MAP2K3,DNAJC3,PSMD7,PTBP1,PTGFRN,TWF1,PTMS,PTPRK,RAB27B,RAD23B,RAP1B,RBMS1,REV3L,RPA1,RPA2,RPN2,RPS17,RRM2,SARS,SBF1,SCD,CXCL11,SDCBP,SRSF7,SH3GL1,SKP2,SLC12A2,SLC12A4,SMARCA1,SMN1,SMN2,SNAI1,FSCN1,SOD2,SON,SOX4,SOX12,SP4,UAP1,SRPRA,STAU1,STRN,SYPL1,TAF4B,TAPBP,DYNLT3,TFDP1,TGFA,TGFBI,TGM2,THBS1,KLF10,TIAL1,SEC62,TNFAIP2,TP53,TPBG,TPI1,TRIO,TSPYL1,PHLDA2,UBE2D3,UBE2H,UGDH,VIM,WNT5A,XPO1,YWHAE,YWHAZ,ZFPL1,MKRN3,ZNF200,ZFAND5,PTP4A1,IFRD2,REEP5,USP5,FXR1,SLC7A5,NCOA3,TRRAP,PICALM,CDC7,STX7,YBX3,NOP14,SOCS1,STX16,BECN1,SNX3,EED,ADAM9,NAPG,TNFRSF10B,PEX11B,SUCLA2,CREG1,PER2,ST3GAL5,CDC123,EIF2B4,PRPF4B,P4HA2,SOCS3,BAZ1B,GPRC5A,CNOT9,CCNE2,SLC33A1,LRRFIP1,VAPA,CCPG1,PNMA1,PDLIM7,TRIP12,GTF3C4,B4GALT5,VAMP3,SLC9A3R2,ZNF264,ROCK2,SLC4A7,FXR2,TBPL1,MPDU1,TMEM59,BAG4,H6PD,PREPL,SH3PXD2A,IQCB1,UBE3C,JADE3,BMS1,CEP350,ZBTB39,TBC1D4,TELO2,JOSD1,TANK,TOM1,SAE1,ARPC5,G3BP1,MBNL2,TRIM2

		8,RBM5,LHFPL2,EIF1,PLIN3,ZMPSTE24,STUB1,,TUBA1B,TUBB3,TUBB4B,SCML2,SEC24B,CFDP1,C1D,TIMM17A,SEC23A,GLRX3,TM9SF1,MRPL28,USP16,PAIP1,PAICS,ERLIN1,CELF1,GNA13,PNMA2,PLEKHO2,TET1,CPEB4,WDR82,SLC35G2,NDFIP1,SLC38A1,NDEL1,TXNDC5,URM1,MAP1LC3B,FAHD1,ADPGK,RAB34,MRO,KREMEN1,QRFPR,WDR75,UTP15,FAM96A,SETD3,LRRC8C,FYTTD1,RNF135,PCGF5,NIFK,MSANTD4,HHIPL1,LCOR,MAK16,CBX2,LMNB2,ADO,PPP1R15B,FAM104A,C8orf76,ZNRF1,STRIP1,ZCRB1,DOCK7,ITPRIP,SELENOI,RRP36,KBTBD6,SEC11C,RFT1,MTDH,SLC38A5,NT5C1B,SFXN1,CAVIN3,PRRT2,LARP4,ADPRHL1,CEP72,BTBD7,N4BP2,CNDP2,WDR12,EXOC2,MBNL3,UTP6,KDM3A,CAND1,USE1,NDUFA12,DHX33,C5orf15,C16orf62,RARS2,AVEN,REXO4,PHTF2,SLC4A10,SCYL1,REXO1,GATAD2B,KIDINS220,NUFIP2,MIB1,TAOK1,NCEH1,SEMA6A,,PCDH10,SHROOM3,LRRN1,USP37,TLDC1,EPG5,ANKRA2,RPRD1B,SCAF1,ELOVL5,SPCS3,ATL2,PAPD5,GIGYF1,COPS7B,S100PBP,FNDC3B,CDCP1,MRPS5,VPS33A,MARCKSL1,DDX50,CAMKV,GGCT,DERL1,FYCO1,EFL1,AKIRIN1,DHX40,YRDC,NOL9,ALG9,RNF122,LPCAT1,SIKE1,EDC3,RUBCNL,ZXDB,MOSPD2,PPTC7,ITPRIPL2,SLFN5,MIER3,CCDC71L,DHX36,FBXO45,TMEM192,CNPY4,LCLAT1,ZDHHC20,ELMOD2,OSBPL8,UHRF2,WDFY2,MOGAT1,THEM4,IP6K3,IKBIP,JDP2,LSM12,ZNF543,LYPLAL1,TPRG1L,PPARGC1B,GRPEL2,HINT3,NACC2,PTRH1,KDELC2,CPNE8,HNRNPA1L2,ZSCAN29,DPY19L3,PHF13,FAM81B,BMT2,FAM91A1,LIN28B,GCSAM,TAB3,GATC,GXYLT1,CLEC4D,IFNE,MTX3,TICAM2,KRT77,MIA3,PTAR1,SKIDA1,GOLGA8B,TOMM5,TMEM41B,MZT1,GPR75-ASB3,HCC,MAP1LC3B2,GTF2H2C,MTRNR2L10
6	hsa-mir-423-3p	ADCY1,SLC25A6,NUDT2,AKT2,ALAS1,FAAP100,KLHL15,YIPF5,PUS3,AMMECR1L,CEP78,USMG5,ZNF503,DGCR6L,MICALL1,KIAA1671,EAF1,CCDC32,RNF185,ZNF561,MRFAP1,LARP4,LYPD1,UBE2J2,ZFYVE27,LRR1,IRGQ,C6orf141,NACC2,RPP25L,MIB2,ZNF420,C22orf42,ZFPM1,POLR3H,TXLNA,B4GALNT3,NOTO,POTEG,LURAP1,POTEM,TATDN2,URB2,NUP58,LRIG2,DDX46,POM121,BCL2L11,SLC25A15,SF3B4,UBE4B,SCML2,KDELR1,HNRNPA0,YWHAQ,GCN1,KIF2C,PAPD7,HNRNPUL1,APP,KIF1A,CALR,CCNT2,CDK7,CDKN1A,AP2M1,COPA,CRK,DPYSL3,EEF1A1,EIF4B,EIF5A,ESRRA,ETF1,EWSR1,F2,FASN,FLII,FLOT2,KDSR,XRCC6,GLDC,GPS1,H1F0,HDLBP,HLAC,HOXD13,HSPA1B,IGF2R,INCENP,IRAK1,MCM2,MAP3K1,MAP3K9,CYTB,MTHFR,MYB,NUBP1,NPTX2,SLC11A2,PA2G4,PABPC3,PDCL,PEPD,PFKL,PKM,PML,POLR2L,PPP2R1B,MAPK3,PSMB5,PSMD1,PSMD8,PTMA,PTMS,PTPRK,RANBP2,RBBP4,UPF1,ROBO1,RPL3,RPL4,RPL26,RPLP1,MRPL12,RPS2,RXRA,ATXN7,SET,SRSF1,SHMT2,SMARCA2,SMARCD2,SPTBN1,SRPRA,TBCE,TLE1,TRPM2,UQCRB,VEGFA,NSD2,MOGS,SMC1A,NAA10,HIST1H3D,TAGLN2,RRP1,EIF3B,EIF3C,SUCLG1,BAZ1B,ATP6V0D1,HGS,LPAR2,ARHGEF2,MTA2,TCEAL1,FADS2,NTN1,RPH3AL,NPEPPS,PUM1,PELP1,COMMD9,RBM15B,SAC3D1,STOML2,IER3IP1,REEP2,RTCB,DDX56,FBXL19,NDE1,CHCHD3,C1orf56,AKIRIN2,LARP1B,MRPS18A,SETD5,FEM1A,LYPLA2,SPEN,CNOT1,ZC3H4,NUP210,SZT2,SF3B3,SF3B1,QPRT,LRRC8B,SUZ12,TNPO3,ZNF324,LSM4,SH2B1,APPL1,RAI14,ABHD12,NARF,AGO1,PABPC1,CYFIP2,WNK1,CENPM,METTL16,PTCD2,MCMBP,KIAA0319L,SLC30A6,FRMD4A,ARFGAP1,FOXJ2,PPAN,TMEM9B,NAT14,SELENON,ATP8B2,KIDINS220,CIP2A,FBRSL1,TBC1D22B,SFMBT2,RAP2C,WIZ,RBSN,MFSD14A,C6orf106,TBC1D15,NT5DC2,RNF213

7	hsa-mir-4524a-5p	NR6A1,GNAT1,GPR27,HNRNPA1,HSPA8,KIF5B,KPNA3,SMAD7,MYO5A,OCRL,ORC4,CDK17,PHKA1,PIK3R1,PLAG1,PPM1A,PPP2R5C,PRKAR2A,MAPK8,PTPRD,MAP4K2,REL,RS1,SALL1,ATXN2,SKI,SLC2A3,SNRPB2,SNTB2,SRPRA,MAP3K7,PPP1R11,TEF,TFAP2A,TLL1,UGT2B4,VEGFA,WEE1,RECK,CUL3,CBX4,CASK,SLC25A12,NAPG,MTMR3,CCNE2,HMGN3,GLP2R,KIF23,SOCS5,N4BP1,BZW1,TSC22D2,TLK1,DMTF1,AKT3,ACTR2,CTDSPL,LANCL1,BTN3A3,NUP50,ARPP19,SEC24A,PNPLA6,PRSS21,PRDM4,ZNF507,TRAK1,SETD1B,TRIM35,KANK1,NNT,CD2AP,TMEM245,PISD,MOB4,SZRD1,WIPI2,SLCO3A1,ZBTB44,HCFC2,SEC61A1,PSAT1,ASCC1,ZNF691,RSL24D1,DNAJC10,USP53,CNNM2,CDCA4,RIF1,SBNO1,TMEM100,PI4K2B,RFK,PNRC2,CDC37L1,SLC30A6,ASH1L,CYP26B1,ASAH2,AP2B1,ATP5G3,CCND1,CA8,CANX,CAPZA2,CCND2,CCNE1,CCNT1,CDC25A,CHEK1,COX6B1,CREBL2,CRK,DDX3X,DECR1,EFNB2,EREG,EXT1,FGF2,DCAF17,ZNF436,VOPP1,C1orf21,GSG1,BCL2L12,ZNRF3,UTP15,USP48,DCTN5,PARD6B,CBX2,ZC3H10,ZNF622,SESTD1,YTHDC1,TMEM44,SCAMP4,RAB3IP,OSCAR,KLHDC7A,UBR3,DYNLL2,SREK1,CACUL1,HNRNPA1L2,CREBRF,CNKSR3,SPRED1,ZNF367,ZNF449,CCDC83,SLC16A9,FOXK1,ZNRF2,ZNF620,UBN2,GAS2L3,RNF149,C3orf38,LURAP1L,MMAB,XKR7,SFT2D2,FAM229B,ZNF704,ASAH2B,HSPE1MOB4,CDC42SE2,RTN4,RALGAPB,ACTR3B,ODF2L,NUFIP2,TAOK1,KIAA1456,DLGAP3,PLEKHA1,ZMAT3,RAPH1,WNK3,CHAC1,YRDC,ZFHX4,ZC3H14,ATAD5,L2HGDH,FBXL18
8	hsa-mir-486-5p	SMAD2,SERPINE1,PCCA,LTBP2,FOXO1,FPR1,H3F3B,HMGA1,HPGD,IGSF3,ID4,IGF1R,ARF6,ARHGAP5,CD40,CDK4,DOCK3,FBN1,CENPN,YAE1D1,FAM217B,CCDC14,TM4SF20,SPRTN,CMSS1,UTP4,UBASH3B,ZIC5,SAPCD2,BTF3L4,PIM1,PIK3R1,PTEN,SNAI1,SP4,HAT1,NRP2,MBD4,CLDN10,CRIPT,BAG2,G3BP2,ABCF2,BASP1,OLFM4,ZNF460,TMED1,CIT,SEC23IP,SEL1L3,CADM1,FOXP1,UBE2S,DCTN4,FAM46A,RBM22,RCOR3,TTC8,MACROD2,METTL27,DENND5B,ZNRF2,ZDHHC20,EPGN,RBM12B,ZNF286B

Spplementary Table 3: List of significant pathways enriched with targets of eight significant DEMs predicted with KEGG database at mirNet

Sr. No.	KEGG Pathway	p Value
1	RNA transport	4.58E-07
2	Pathways in cancer	7.29E-07
3	Colorectal cancer	1.03E-06
4	Neurotrophin signaling pathway	4.45E-06
5	Cell cycle	4.73E-06
6	HTLV-I infection	7.18E-06
7	Pancreatic cancer	0.0000107
8	Prostate cancer	0.0000107
9	Renal cell carcinoma	0.0000147
10	Focal adhesion	0.0000258
11	mTOR signaling pathway	0.0000258
12	p53 signaling pathway	0.0000607
13	Epstein-Barr virus infection	0.0000708
14	Chronic myeloid leukemia	0.0000822
15	Non-small cell lung cancer	0.000087
16	Influenza A	0.000177
17	Endometrial cancer	0.000189
18	Insulin signaling pathway	0.000213
19	Adherens junction	0.000242
20	Apoptosis	0.000355
21	Jak-STAT signaling pathway	0.000355
22	Glioma	0.000446
23	Chagas disease (American trypanosomiasis)	0.000505
24	Toxoplasmosis	0.000505
25	Bacterial invasion of epithelial cells	0.000758
26	ErbB signaling pathway	0.000758
27	Hepatitis C	0.000821
28	Melanoma	0.000821
29	Protein processing in endoplasmic reticulum	0.000821
30	Progesterone-mediated oocyte maturation	0.000834
31	Acute myeloid leukemia	0.000848
32	Measles	0.00114
33	T cell receptor signaling pathway	0.00114
34	Regulation of actin cytoskeleton	0.00142
35	Small cell lung cancer	0.00187
36	Osteoclast differentiation	0.00233
37	Bladder cancer	0.00331
38	B cell receptor signaling pathway	0.00354

39	Salmonella infection	0.0044
40	Wnt signaling pathway	0.0068
41	Pathogenic Escherichia coli infection	0.00732
42	Synaptic vesicle cycle	0.00819
43	Shigellosis	0.00836
44	Toll-like receptor signaling pathway	0.0177
45	Amyotrophic lateral sclerosis (ALS)	0.0218
46	Leukocyte transendothelial migration	0.0231
47	Type II diabetes mellitus	0.0265
48	Pyrimidine metabolism	0.03
49	RIG-I-like receptor signaling pathway	0.0317
50	VEGF signaling pathway	0.0388
51	Oocyte meiosis	0.0396
52	Vibrio cholerae infection	0.0437
53	RNA degradation	0.0481

Supplementary Table 4: List of genes having an overlap in differentially expressed genes (as obtained from gene expression array data) and the gene targets (as predicted by miRNet) of the eight DEMS in CLL

Sr. No.	Genes	Fold change CLL Vs. Healthy
1	ARG1	3.36
2	ARHGAP5	2.13
3	AURKA	-3.02
4	AURKB	-4.27
5	CADM1	-2.86
6	CCR9	-7.68
7	CDC20	-4.01
8	CDC25A	-3.23
9	CDCA3	-2.75
10	CENPM	-4.16
11	CEP55	-3.36
12	CHEK1	-2.21
13	CTDSPL	-2.59
14	CTLA4	4.72
15	DIAPH3	-2.36
16	DMD	2.38
17	DOCK7	2.91
18	DSP	-6.16
19	DTL	-2.5
20	EDN1	2.07
21	EPHA4	-3.1
22	EYA2	-4.91
23	FITM2	-2.33
24	FNDC3B	-3.21
25	FOXD1	-3.5
26	FRZB	-3.7
27	FSTL1	-2.24
28	GAB1	2.04
29	GCSAM	-3.35
30	GLDC	-7.78
31	GNB4	-2.28
32	GPT2	2.31
33	IGF1	-4.61
34	IL6	-5.7
35	INPP5F	2.12

36	ITGB3	-2.52
37	JUP	-4.14
38	KCNN3	-3.38
39	KIAA1671	2.17
40	KIF11	-2.17
41	KIF14	-2.35
42	KIF2C	-2.84
43	LAIR1	-2.45
44	LIMCH1	2.49
45	LPL	4.03
46	LRRC20	-2.24
47	LRRN1	2.33
48	MACROD2	-3.08
49	MET	-3.9
50	MMP9	3.51
51	MXI1	2.14
52	MYC	-2.56
53	MYO1D	-2.9
54	NAMPT	2.39
55	NRGN	-3
56	NRSN2	2.03
57	NT5DC2	-4.01
58	NT5E	-3.87
59	PHGDH	-3.16
60	PIK3C2B	-2.84
61	PLAG1	-2.59
62	PLK1	-2.81
63	PMAIP1	2.13
64	PNMA2	3.87
65	PODXL	-2.62
66	PPA1	-2.12
67	PPFIBP1	-4.33
68	PRDM1	-2.93
69	PSAT1	-3.94
70	PTPRK	-5.79
71	PYCR1	-2.78
72	RAPH1	-2.8
73	REEP2	2.66
74	RHBDF2	-2.44

75	RRM2	-4.26
76	RUNX2	-2.41
77	SAPCD2	-2.72
78	SEC11C	-2.66
79	SEC24A	-2.2
80	SLC7A11	-2.46
81	SLC7A5	-2.97
82	SMAD3	-2.65
83	SOBP	2.18
84	SPCS3	-2.94
85	SYNE2	2.41
86	TGFBR3	3.59
87	TLE4	2.04
88	TMEM136	2.16
89	TNFSF9	2.39
90	TRIP13	-2.95
91	TUBB3	-2.13
92	TXNDC5	-6.23
93	UBASH3B	3
94	YBX3	2.37
